# A de novo peroxidase is also a promiscuous yet stereoselective carbene transferase

**DOI:** 10.1073/pnas.1915054117

**Published:** 2020-01-02

**Authors:** Richard Stenner, Jack W. Steventon, Annela Seddon, J. L. Ross Anderson

**Affiliations:** ^a^School of Biochemistry, University of Bristol, BS8 1TD Bristol, United Kingdom;; ^b^Bristol Centre for Functional Nanomaterials, HH Wills Physics Laboratory, University of Bristol, BS8 1TL Bristol, United Kingdom;; ^c^BrisSynBio Synthetic Biology Research Centre, University of Bristol, BS8 1TQ Bristol, United Kingdom;; ^d^School of Physics, HH Wills Physics Laboratory, University of Bristol, BS8 1TL Bristol, United Kingdom

**Keywords:** de novo protein design, enzyme design, carbene transfer, biocatalytic ring expansion, biocatalysis

## Abstract

By constructing an in vivo-assembled, catalytically proficient peroxidase, C45, we have recently demonstrated the catalytic potential of simple, de novo-designed heme proteins. Here, we show that C45’s enzymatic activity extends to the efficient and stereoselective intermolecular transfer of carbenes to olefins, heterocycles, aldehydes, and amines. Not only is this a report of carbene transferase activity in a completely de novo protein, but also of enzyme-catalyzed ring expansion of aromatic heterocycles via carbene transfer by any enzyme.

## Significance

While the bottom-up design of enzymes appears to be an intractably complex problem, a minimal approach that combines elementary, de novo-designed proteins with intrinsically reactive cofactors offers a simple means to rapidly access sophisticated catalytic mechanisms. Not only is this method proven in the reproduction of powerful oxidative chemistry of the natural peroxidase enzymes, but we show here that it extends to the efficient, abiological—and often asymmetric—formation of strained cyclopropane rings, nitrogen–carbon and carbon–carbon bonds, and the ring expansion of a simple cyclic molecule to form a precursor for NAD+, a fundamentally important biological cofactor. That the enzyme also functions in vivo paves the way for its incorporation into engineered biosynthetic pathways within living organisms.

Despite the significant advances in protein design, there still remain few examples of de novo enzymes constructed from bona fide, de novo protein scaffolds that both approach the catalytic efficiencies of their natural counterparts and are of potential use in an industrial or biological context (1–10). This reflects the inherent complexities experienced in the biomolecular design process, where approaches are principally focused on either atomistically precise redesign of natural proteins to stabilize reaction transition states (1–4) or imprinting the intrinsic chemical reactivity of cofactors or metal ions on simple, generic protein scaffolds (5–10); both can be significantly enhanced by implementing powerful, yet randomized, directed evolution strategies to hone and optimize incipient function (11, 12). While the latter approach often results in de novo proteins that lack a singular structure (6, 13, 14), the incorporation of functionally versatile cofactors, such as heme, is proven to facilitate the design and construction of de novo proteins and enzymes that recapitulate the function of natural heme-containing proteins in stable, simple, and highly mutable tetrahelical chassis (termed maquettes) (15–17). Since the maquettes are designed from first principles, they lack any natural evolutionary history, and the associated functional interdependency that is associated with natural protein scaffolds can be largely circumvented (15).

**Fig. 1.**
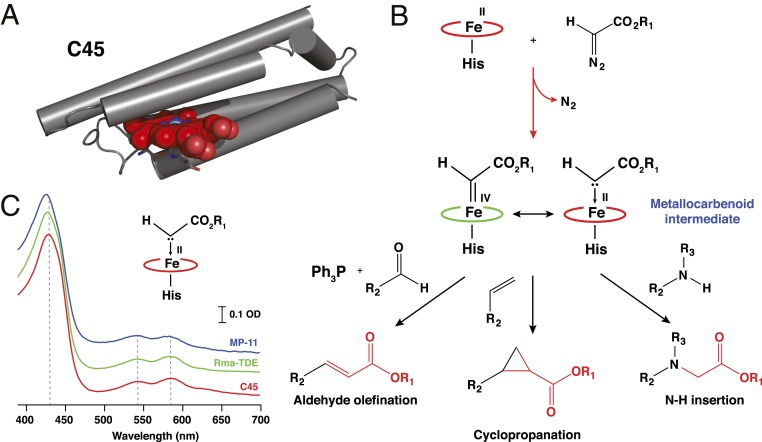
Metallocarbenoid formation and its reactive potential within a de novo-designed *c*-type cytochrome maquette, C45. (*A*) Single snapshot from a 1-μs molecular-dynamics simulation of the C45 maquette (6). (*B*) Formation and potential reactivity of a heme-based metallocarbenoid intermediate, illustrating aldehyde olefination, olefin cyclopropanation, and amine N–H insertion reactions. (*C*) UV/visible EDA-treated C45 (red), engineered *Rma-*TDE (green), and MP-11 (blue) obtained by rapid mixing experiments in a stopped-flow spectrophotometer. The putative metallocarbenoid species was generated by mixing 2.5 mM EDA with 7.5 μM ferrous heme protein in 100 mM KCl and 20 mM CHES, pH 8.6 (10% EtOH).

We have recently reported the design and construction of a hyperthermostable maquette, C45, that is wholly assembled in vivo, hijacking the natural *Escherichia coli* cytochrome *c* maturation system to covalently append heme onto the protein backbone (Fig. 1*A*) (6). The covalently linked heme C of C45 is axially ligated by a histidine side chain at the proximal site, and it is likely that a water molecule occupies the distal site, analogous to the ligation state of natural heme-containing peroxidases (18) and metmyoglobin (19). Not only does C45 retain the reversible oxygen-binding capability of its ancestral maquettes (6, 15, 16), but it functions as a promiscuous and catalytically proficient peroxidase, catalyzing the oxidation of small molecules, redox proteins, and the oxidative dehalogenation of halogenated phenols with kinetic parameters that match and even surpass those of natural peroxidases (6).

It has been recently demonstrated that several natural heme-containing proteins and enzymes [e.g., cytochromes P450 (20), globins (21), and cytochrome *c* (22)] are capable of accessing many chemistries intrinsic to the heme cofactor, not all of which are essential to life-supporting biological roles. These reported activities are mostly dependent on accessing hypothetical heme-based carbene and nitrene intermediates (23) analogous to the oxene intermediates, observed in the catalytic cycles of heme-containing peroxidases and oxygenases (18). Several groups have now reported examples of natural and engineered hemoproteins that catalyze cyclopropanations (20, 24–31), C–H insertions (32–35), carbonyl olefinations (36, 37), N–H insertions (21, 38), C–H amination/amidation (39, 40), boron alkylations (41), aziridinations (42), and C–Si bond formations (22), most of which have been exposed to several rounds of directed evolution to improve stereoselectivity and product yield. Excluding the aziridation reactions, an electrophilic metallocarbenoid intermediate (Fig. 1*B*) is hypothesized to be responsible for carbene transfer to a suitable nucleophile (e.g., olefin) (23, 43) in these reactions. We therefore reasoned that if the metallocarbenoid intermediate could be detected spectroscopically in C45, then the de novo enzyme may function as a promiscuous carbene transferase, catalyzing a range of important and challenging organic transformations.

## Results and Discussion

### Generation of Reactive Metallocarbenoids in the De Novo-Designed C45 and an Engineered Cytochrome *c* (*Rma*-TDE).

**Fig. 2.**
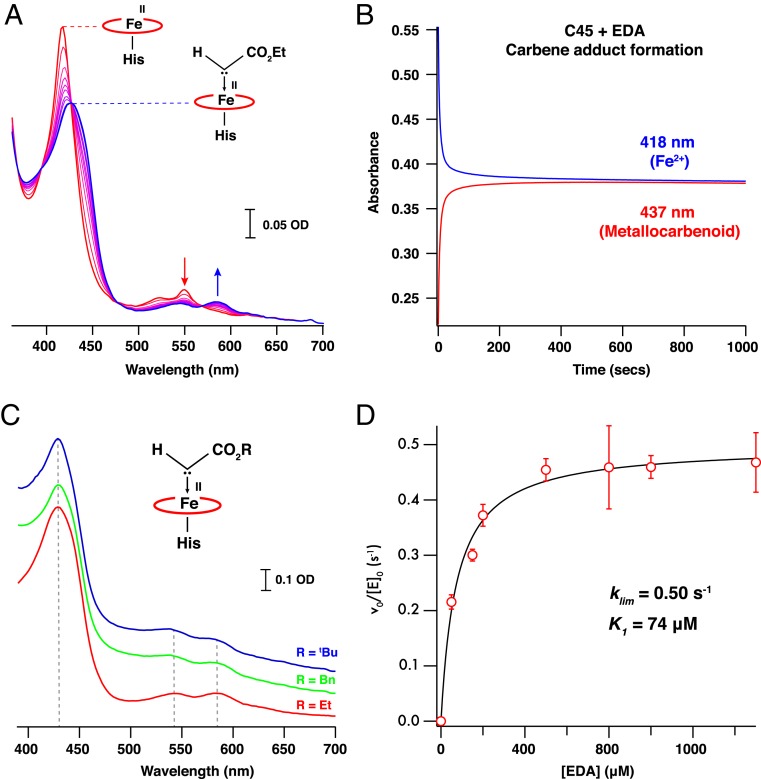
Stability and reactivity of the metallocarbenoid:C45 intermediate. (*A*) Time course of electronic spectra recorded following rapid mixing of ferrous C45 (red) with EDA in 10% EtOH at 5 °C. The appearance of the metallocarbenoid intermediate (blue) is concomitant with the disappearance of the ferrous C45 spectrum. Spectra presented were recorded 1, 2, 4, 6, 8, 10, 15, 20, 50, and 100 s after mixing. (*B*) Metallocarbenoid formation and stability in the absence of styrene substrate. Single-wavelength traces represent the time course of ferrous C45 (418 nm; blue; 7.5 μM protein, 10% EtOH) and metallocarbenoid:C45 adduct (437 nm; red) following rapid mixing of ferrous C45 with 500 μM EDA at 5 °C. Once formed, the metallocarbenoid:C45 adduct persists for the duration of the experiment (1,000 s). (*C*) Electronic spectra of metallocarbenoid intermediates formed between ferrous C45 and EDA, BnDA, and ^*t*^BuDA following rapid mixing in the stopped-flow apparatus. (*D*) EDA-concentration-dependent formation of the C45 metallocarbenoid adduct. Kinetic data were recorded by using a stopped-flow spectrophotometer and analyzed as described in *Experimental Details*. The limiting rate constant (*k*_*lim*_) and pseudo-Michaelis constant (*K*_*1*_) for metallocarbenoid formation are 0.50 s^−1^ and 74 μM, respectively. Data were collected in triplicate, and error bars represent the SD.

To spectroscopically isolate the metallocarbenoid intermediate, we rapidly mixed ferrous C45 with the carbene precursor, ethyl diazoacetate (EDA), at 5 °C in a stopped-flow spectrophotometer. Concomitant with the disappearance of the ferrous C45 spectrum was the appearance of a spectroscopically distinct species over 60 s, with a red-shifted Soret peak at 429 nm and broad Q bands centered at 543 and 586 nm (Figs. 1*C* and 2*A*). Under these conditions, this spectrum persisted for 1,000 s with almost no degradation (Fig. 2*B*), and it was spectroscopically consistent with spectra reported for experimentally produced carbene:iron porphyrin complexes (44). For our proposed C45 metallocarbenoid intermediate, the cold conditions on rapid mixing proved essential to spectroscopic observation, as room-temperature experiments resulted in spectra indicative of carbene-induced heme degradation, consistent with a mechanism proposed by Arnold and colleagues (45). It was also necessary to employ an ethanol:water mixture to ensure EDA solubility and stability for generating the putative intermediate in the stopped flow at 5 °C, and we observed an identical, long-lived spectrum at ethanol concentrations between 10 and 50% (*SI Appendix*, Fig. S1). While ethanol concentrations above 50% are generally avoided in buffered protein solutions due to denaturation, small helical bundles such as C45 can readily tolerate such aqueous:organic mixtures (5, 6), retaining structure and catalytic activity. Substituting EDA for benzyl-diazoacetate (BnDA) and *tert*-butyl-diazoacetate (^*t*^BuDA) also resulted in the appearance of intermediates with near-identical spectroscopic properties to the EDA-generated C45 species (Fig. 2*C*), demonstrating the intrinsic flexibility of the active site in accommodating bulky diazoacetate substituents. We were subsequently able to generate and measure mass spectra of these putative intermediates using mild ionization/near-native conditions using positive electrospray ionization mass spectrometry (ESI-MS) (*SI Appendix*, Fig. S2). Mass spectra of the EDA-, BnDA-, and ^*t*^BuDA-generated metallocarbenoids of C45 exhibited the mass differences expected for the adducts, further confirming the nature of the species generated under these conditions.

Under identical conditions to C45, we individually mixed ferrous microperoxidase-11 (MP-11) and an engineered cytochrome *c* from *Rhodothermus marinus* (46) (*Rma*-TDE) with EDA, the latter with an established carbene transferase activity with respect to silanes (46). Near-identical spectra to the putative metallocarbenoid C45 were obtained (Fig. 1*C*), though significant differences were apparent between our spectra and those reported for the metallocarbenoid complex of *Rma-*TDE (46). Since our spectra were collected under more rigorously anaerobic conditions than the previous study (stopped-flow spectrophotometer housed in an anaerobic glove box; <5 parts per million [ppm] O_2_) and at a lower temperature, we postulate that the reported ultraviolet (UV)/visible spectra instead represent an alternative, yet currently unidentified, species. In contrast to C45, *Rma-*TDE exhibits a lag phase during the formation of the putative intermediate, with noticeably slower kinetics compared to C45 under the same experimental conditions. To probe this further, we measured the EDA concentration-dependent kinetics of the putative metallocarbenoid formation for C45 and *Rma-*TDE (Fig. 2*D* and *SI Appendix*, Fig. S3). C45 exhibited both a higher limiting rate constant (*k*_*lim*_) for metallocarbenoid formation and lower pseudo-Michaelis constant (*K*_*1*_) for EDA (*k*_*lim*_ = 0.50 s^−1^; *K*_*1*_ = 74 μM) compared to *Rma-*TDE (*k*_*lim*_ = 0.14 s^−1^; *K*_*1*_ = 490 μM), demonstrating that not only does C45 bind EDA more rapidly than *Rma-*TDE, but it likely has a significantly higher affinity for the carbene precursor.

### Cyclopropanation Activity of C45 and *Rma-*TDE.

**Fig. 3.**
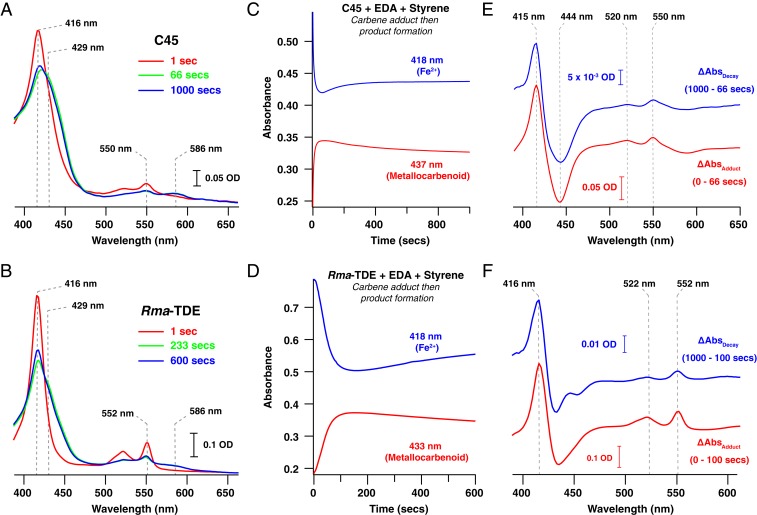
Reactivity of the metallocarbenoid intermediates of C45 and *Rma*-TDE. (*A* and *B*) Electronic spectra of C45 (*A*) and *Rma*-TDE (*B*) recorded after rapid mixing of ferrous heme protein (7.5 μM; red trace) with EDA (500 μM) and styrene (3 mM) at 5 °C in the stopped-flow spectrophotometer. Green traces correspond to spectra recorded at time points where the maximum quantity of metallocarbenoid was accumulated; blue traces correspond to the final spectra recorded in the experiments. (*C* and *D*) Metallocarbenoid formation and decay of C45 (*C*) and *Rma*-TDE (*D*) in the presence of styrene. Single-wavelength traces represent the time course of ferrous heme protein (blue traces) (7.5 μM protein) and metallocarbenoid adduct (red) following rapid mixing of ferrous heme protein with 500 μM EDA and 3 mM styrene at 5 °C. (*E* and *F*) Electronic difference spectra highlighting the spectroscopic changes associated with metallocarbenoid formation and decay for C45 (*E*) and *Rma*-TDE (*F*). The lower, red traces demonstrate the spectroscopic changes that occur during the formation of the metallocarbenoid, which are identical to those observed during its subsequent decay (upper, blue traces). These indicate reformation of the initial ferrous species after carbene insertion and after EDA is exhausted.

We subsequently investigated the ability of C45 to act as an active carbene transferase in the cyclopropanation of styrene, commonly used as an acceptor for heme protein-derived metallocarbenoids (20). To confirm the identity of the spectroscopically isolated species as the putative metallocarbenoid intermediate, we rapidly mixed ferrous C45 with 100 μM EDA and 3 mM styrene at 5 °C in a stopped-flow spectrophotometer (Fig. 3). Under these conditions, the same putative intermediate spectrum appeared over 60 s, but then decayed slowly to the starting ferrous C45 spectrum, consistent with the proposed mechanism of heme-catalyzed carbene transfer to the olefin, in which there is no net transfer of electrons from the heme (47, 48). No other spectroscopically distinct species was observed during this experiment. The putative metallocarbenoid species of the engineered *Rma-*TDE displayed analogous behavior to C45 in the presence of styrene, with the disappearance of the metallocarbenoid intermediate and concomitant reappearance of the ferrous *Rma-*TDE spectrum (Fig. 3). In contrast, the aerobically generated species prepared by room-temperature mixing of *Rma-*TDE with EDA did not exhibit such behavior, and there was no detectable cyclopropanation activity. Given these data, we assigned the spectroscopically distinct species we described above to the C45 and *Rma-*TDE metallocarbenoid intermediates. It should be noted that, at this time, we cannot definitively assign these spectra as either the nonbridging metallocarbenoid species observed by Lewis et al. (46) or the porphyrin-bridging species observed by Hayashi et al. (49) in the crystal structures of engineered cytochrome *c* or *N*-methylhistidine–ligated myoglobin variant [Mb(H64V,V68A)], respectively. However, given the identical nature of the C45 and *Rma-*TDE spectra, it would seem likely that the spectra obtained represent the nonbridging ligation observed in the *Rma-*TDE Me-EDA crystal structure (46). Additionally, while the rate of metallocarbenoid intermediate and subsequent product formation appeared relatively low in our stopped-flow experiments, it is worth noting that the selected conditions were necessary for maximizing the quantity of intermediate in the stopped-flow apparatus and that subsequent activity assays were carried out at higher substrate concentrations (both EDA and styrene), higher temperature, and lower ethanol concentrations. This would undoubtedly lead to higher reaction rates than those presented in the stopped-flow data here.

**Fig. 4.**
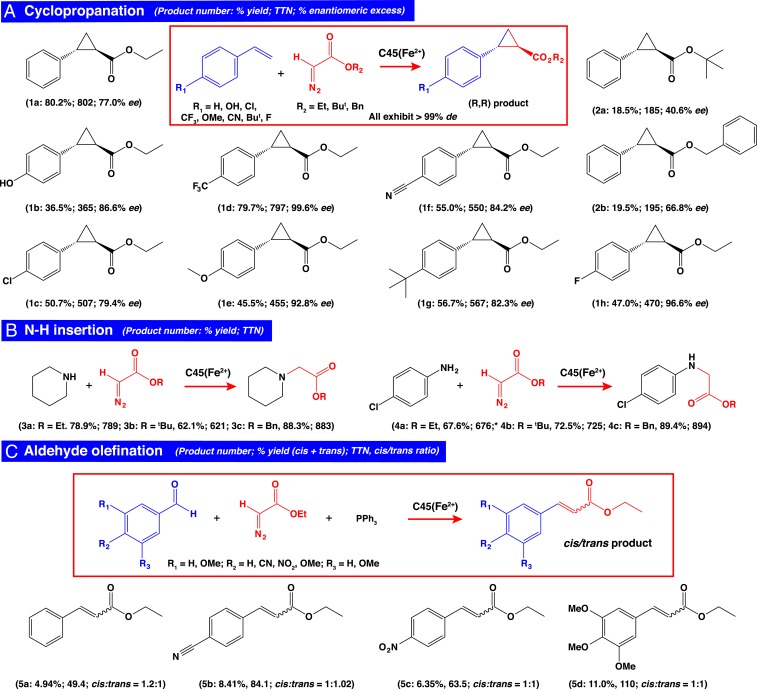
Carbene transferase activity of C45. (*A*) Cyclopropanation of substituted styrenes catalyzed by C45. TTNs and % *ee* for each combination of ferrous C45 with *para*-substituted styrenes and functionalized diazoacetates are shown. Only the (*R,R*) cyclopropanated product is displayed in the reaction scheme, representing the dominant product in all cases. All reactions were carried out with 0.1% catalyst loading (10 μM C45) at the following concentrations of reagents: 10 mM sodium dithionite, 10 mM diazo compound, and 30 mM substituted styrene in 100 mM KCl, 20 mM CHES (pH 8.6), and 5% EtOH/H_2_O. (*B*) N–H insertion of primary and secondary amines catalyzed by C45. Only the monofunctionalized product of the *p*-chloroaniline insertion reaction is shown, and the corresponding TTN is calculated based on the yield of both monosubstituted and disubstituted products. All reactions were carried out with 0.1% catalyst loading (10 μM C45) at the following concentrations of reagents: 10 mM sodium dithionite, 10 mM EDA, and 30 mM amine substrate in 100 mM KCl, 20 mM CHES (pH 8.6), and 5% EtOH/H_2_O. (*C*) Olefination of substituted benzaldehydes catalyzed by C45. All reactions were carried out with 0.1% catalyst loading (10 μM C45) at the following concentrations of reagents: 10 mM sodium dithionite, 10 mM PPh_3_, 10 mM EDA, and 30 mM substituted benzaldehyde in 100 mM KCl, 20 mM CHES (pH 8.6), and 5% EtOH/H_2_O.

Cyclopropanation reactions were initiated at 5 °C under anaerobic conditions and at low ethanol concentrations (5% EtOH) and were subsequently allowed to warm to room temperature. We then analyzed the cyclopropanation activity of C45 and hemin, respectively, when mixed with EDA and styrene by chiral high-performance liquid chromatography (HPLC) and liquid chromatography–mass spectrometry (LC-MS). Hemin exhibited little cyclopropanation activity, and, under the reaction conditions employed here, only unreacted EDA was observed. However, analysis of the C45-catalyzed reaction revealed the presence of 2 peaks in the chiral chromatogram at 7.23 and 9.00 min, corresponding to the (*R,R*) and (*S,S*) enantiomers of the ethyl 2-phenylcyclopropane-1-carboxylate (Et-CPC; **1a**) product (*SI Appendix*, Figs. S4 and S5). Following base hydrolysis of the Et-CPC to the corresponding acid (2-phenylcyclopropane-1-carboxylic acid; CPC) and further LC-MS and chiral HPLC analysis against commercial standards (*SI Appendix*, Figs. S5 and S6), we determined that the C45-catalyzed cyclopropanation of styrene was highly diastereoselective (>99% *de*), occurred at high yield (80.2%, total turnover number [TTN] = 802, turnover frequency [TOF] = 6.68 min^−1^), and exhibited significant enantioselectivity, with an enantiomeric excess (*ee*) of 77% in favor of the (*R,R*) enantiomer. C45 will accept derivatized diazoacetates and *para*-substituted styrenes as substrates for cyclopropanation, producing products with varying stereoselectivities and yields (**1a**–**h**, **2a**, and **2b**; Fig. 4, Table 1, and *SI Appendix*, Figs. S7–S9). In particular, C45/EDA-catalyzed cyclopropanation of *p*-trifluoromethylstyrene, *p*-methoxystyrene, and *p*-fluorostyrene occurred with exceptional enantioselectivity for the (*R,R*) product (99.6% *ee* for F_3_C-substituted, **1d**; 92.8% *ee* for MeO-substituted, **1e**; and 96.6% *ee* for F-substituted, **1h**). However, ^*t*^BuDA and BnDA provided lower yields and enantioselectivities of products **2a** (18.5% and 40.6% *ee*) and **2b** (19.5% and 66.8% *ee*). We postulate that the observed stereoselectivity exhibited by C45 resulted from the intrinsic asymmetry of the protein scaffold, likely through specific positioning of the reactive carbene adduct, as proposed by Villarino et al. (25) and more recently observed in the crystal structure of the methyl-EDA adduct of the engineered cytochrome *c* (46). Our previous work (6) has demonstrated that C45 exists in a dynamic and conformationally heterogeneous state, at least under the conditions and concentrations used for NMR studies. This suggests that a native-like state is neither required for this type of chemical catalysis in a protein nor for achieving a high degree of stereoselectivity in the metalloprotein-catalyzed cyclopropanation of styrene. Indeed, a conformationally dynamic environment at the distal face of the heme may facilitate access of both the diazoacetate and styrene (46) into the active site, while subsequently providing sufficient space for the olefin to asymmetrically attack the electrophilic metallocarbenoid.

**Table 1.** C45-catalyzed cyclopropanation of styrene and *para*-substituted styrenes by EDA and of styrene by substituted diazoacetates

Product	R1	R2	% Yield (*R,R*)	% Yield (*S,S*)	*de* (*E*)%	*ee* (*R,R*)%	TTN (*R,R*)	Reaction time (min)	TOF/min^−1^
1a	H	Et	80.2 ± 8.0	10.4 ± 1.80	99.9 ± 0.1	77.0 ± 2.8	802	120	6.68
1b	OH	Et	36.5 ± 5.7	2.65 ± 0.67	99.9 ± 0.1	86.6 ± 1.3	365	120	3.04
1c	Cl	Et	50.7 ± 3.3	5.81 ± 0.19	99.9 ± 0.1	79.4 ± 0.6	507	120	4.23
1d	F_3_C	Et	79.7 ± 4.0	0.14 ± 0.04	99.9 ± 0.1	99.6 ± 0.1	797	120	6.64
1e	OMe	Et	45.5 ± 1.7	1.71 ± 0.10	99.9 ± 0.1	92.8 ± 0.2	455	120	3.80
1f	CN	Et	55.0 ± 8.7	4.75 ± 0.92	99.9 ± 0.1	84.2 ± 0.5	550	120	4.58
1g	^*t*^Bu	Et	56.7 ± 1.0	5.52 ± 0.12	99.9 ± 0.1	82.3 ± 0.4	567	120	4.73
1h	F	Et	47.0 ± 0.8	0.83 ± 0.01	99.9 ± 0.1	96.6 ± 0.2	470	120	3.92
2a	H	^t^Bu	18.5 ± 8.1	7.00 ± 1.54	99.9 ± 0.1	40.6 ± 16.5	185	120	1.92
2b	H	Bn	19.5 ± 1.4	4.00 ± 1.41	99.9 ± 0.1	66.8 ± 7.4	195	120	2.09

Values are ± SDs.

### Altering the Stereoselectivity of Styrene Cyclopropanation in C45.

The apparent asymmetry provided by the protein scaffold offers a means by which we can improve subsequent C45-derived maquettes. In fact, small-scale screening of a C45 library generated by successive generations of error-prone PCR (epPCR) and originally created for improved peroxidase activity revealed a variant of C45 (AP3.2) with switched and remarkably high enantioselectivity toward the (*S,S*) product (*ee* = 99%), albeit with a lower yield (41.5%) compared to C45 (*SI Appendix*, Fig. S10). To probe the flipped stereoselectivity and reduced yield exhibited by AP3.2, we purified and characterized the protein both spectroscopically and functionally. AP3.2 exhibited near-identical UV/visible ferric and ferrous spectra to C45 (6) (*SI Appendix*, Fig. S11*A*) and a circular dichroism (CD) spectrum consistent with that expected for a 4-helix bundle maquette (6, 15, 17) (*SI Appendix*, Fig. S11*B*), albeit with reduced secondary structure compared to C45. The 8 mutations to C45 (F11Y/G39S/D48Y/F53S/F83S/G109A/F132S/E133G) destabilized the protein scaffold, with AP3.2 exhibiting a 37 °C drop in the thermal melting transition (*T*_*m*_ = 49 °C) in comparison to C45 (6) (*SI Appendix*, Fig. S11*C*). Given that 3 of these substitutions replaced core, hydrophobic phenylalanines in helical positions with the hydrophilic serine, this was not surprising, especially given that serine has a significantly lower helical propensity than phenylalanine (50). Mapping the AP3.2 mutations onto our computationally generated C45 model (6) (*SI Appendix*, Fig. S11*D*) did not immediately reveal an obvious explanation for the pronounced change in stereoselectivity we observed, as only the F53S mutation was positioned near to the carbene binding site at the distal face of the heme. To examine reactivity in more detail, we generated the AP3.2 metallocarbenoid intermediate by rapid mixing under the same conditions as described for C45, and we observed an identical intermediate spectrum that accumulated on a comparable timescale to C45 (*SI Appendix*, Fig. S12) and, in the presence of styrene, decayed at a comparable rate to C45 with identical associated spectroscopic changes (*SI Appendix*, Fig. S13 *A*–*C*). To test the possibility of an electronic effect playing a role in the suppressed activity of AP3.2, we used redox potentiometry to measure the AP3.2 heme redox potential (*E*_*m*_) (*SI Appendix*, Fig. S13*D*). AP3.2 exhibited a 26-mV negative shift in redox potential with respect to C45 [C45, *E*_*m*_ = −176 mV (6); AP3.2, *E*_*m*_ = −202 mV], indicating slight stabilization of the ferric form, possibly due to increased solvent accessibility at the heme (51). While this shift also indicated that the extent of electron donation to the bound carbene was indeed altered following the mutations, it was relatively modest in comparison to those observed after heme axial ligand substitution in engineered P450s (51, 52). In these cases, the correlation between large redox potential changes and the specific reactivity of the metallocarbenoid is currently unclear, at least experimentally, and increased reactivity resulting from a more electrophilic carbene adduct would be expected to correlate with a more electron-withdrawing, higher-redox potential heme (43). Furthermore, we identified another C45 variant (APR1) from the library with similarly altered redox potential (APR1; *E*_*m*_ = −197 mV; Δ*E*_*m*_ = −21 mV), but with negligible difference in yield or enantioselectivity [yield = 85.6%, TTN = 856, *ee*(*R,R*) = 78.0%; *SI Appendix*, Fig. S14], therefore highlighting that such small differences in *E*_*m*_ likely contribute little to the overall yield of product under these reaction conditions. As is the case for many enzymes improved by random laboratory evolution methodologies (52), it is therefore not unambiguously clear why this altered reactivity and enantioselectivity occurs, and a currently uncharacterized combination of static and dynamic processes likely play a role in the observed catalytic and stereoselective changes. It does, however, demonstrate well the mutability and catalytic potential of the maquette scaffold and will be explored in more depth in future work.

### Benchmarking the Cyclopropanation Activity of C45.

Since there are now several examples of engineered and natural proteins capable of catalyzing carbene transfer, we wished to benchmark the activity of C45 against some notable and well-characterized carbene transferases. To this end, we directly compared the C45-catalyzed cyclopropanation of styrene with EDA against the corresponding reactions with *Rma-*TDE (22, 46) and a double mutant of sperm whale myoglobin, Mb(H64V,V68A) (26) (Table 2 and *SI Appendix*, Figs. S15 and S16). While *Rma-*TDE was engineered toward silane alkylations (22), it forms a structurally characterized carbene adduct and is a *c*-type cytochrome, rendering it the most spectroscopically similar carbene transferase to C45 in the literature. In contrast, the cyclopropanation activity of Mb(H64V,V68A) and other Mb variants toward styrene has been extensively studied and is both high yielding and highly enantioselective for the (*S,S*) product (26). Under identical conditions to the reactions described above for C45, we confirmed that both proteins catalyze the cyclopropanation of styrene, with Mb(H64V,V68A) exhibiting >99% *ee* for the (*S,S*) product (yield = 95.9%, TTN = 959, TOF = 7.99 min^−1^) as per the literature (26) and *Rma-*TDE exhibiting 70.6% *ee* for the (*R,R*) product (yield = 73.5%, TTN = 735, TOF = 6.12 min^−1^). While the yield from the C45-catalyzed reaction (80.2%) did not surpass that of Mb(H64V,V68A), it is among the highest yields observed for the catalytic cyclopropanation of styrene by EDA, surpassing those exhibited by almost all reported P450 (20, 26–30) and LmrR (25) variants, as well as by several engineered myoglobins (24, 26, 30). In addition, *Rma-*TDE exhibited a relatively high yield and enantioselectivity for styrene cyclopropanation, despite being engineered for carbene insertion into Si–H bonds.

**Table 2.** Comparison of enzyme-catalyzed cyclopropanations of styrene by EDA

Enzyme	R1	R2	% Yield (*R,R*)	% Yield (*S,S*)	*de* (*E*)%	*ee* (*S,S*)%	*ee* (*R,R*)%	TTN*	Reaction time, min	TOF/min^−1^
C45	H	Et	80.2 ± 8.0	10.4 ± 1.80	99.9 ± 0.1	ND	77.0 ± 2.8	802	120	6.68
MgB (H64V/V68A)	H	Et	0	95.9 ± 0.5	99.9 ± 0.1	99.9 ± 0.1	ND	959 (*S,S*)	120	7.99
*Rma*-TDE	H	Et	73.5 ± 5.7	12.6 ± 1.0	99.9 ± 0.1	ND	70.6 ± 2.0	735 (*R,R*)	120	6.12
AP3.2	H	Et	0	41.5 ± 4.6	99.9 ± 0.1	99.9 ± 0.1	ND	415 (*S,S*)	120	3.45
APR1	H	Et	85.6 ± 4.3	11.04 ± 4.8	99.9 ± 0.1	ND	78.0 ± 8.5	856 (*R,R*)	120	7.13

Values are ± SDs. ND, not detected.

* Indicates the TTN for the specific defined enantiomeric species.

### N–H Insertion and Carbonyl Olefination Reactions Catalyzed by C45.

With stereoselective carbene transferase activity firmly established for the cyclopropanation of styrene by C45, we explored the substrate promiscuity of C45 toward similar reactions resulting in N–H insertions and carbonyl olefinations. Following reaction conditions identical to those described above for cyclopropanation and using HPLC and LC-MS to identify insertion products, we observed the C45-catalyzed insertion of EDA/^*t*^BuDA/BnDA-derived carbene into the N–H bonds of piperidine and *para*-chloroaniline (**3a**–**c** and **4a**–**c**; Fig. 4, Table 3, and *SI Appendix*, Figs. S17–S21), with single- and double-insertion products resulting from carbene transfer to the primary amine of *para*-chloroaniline when EDA was employed. Notably, C45 achieved high yields for the insertion of BnDA-derived carbene into the piperidine and *para*-chloroaniline N–H bonds, with 88.3% and 89.4%, respectively (TTN = 883 for **3c**; TTN = 894 for **4c**). For carbonyl olefination, the addition of triphenylphosphine to the reaction mixture is necessary to facilitate the formation of a ylide that, through the generation of an oxaphosphetane intermediate, spontaneously collapses to the product (36, 37). We investigated the olefination of benzaldehyde and 3 substituted benzaldehyde variants with differing electron-donating and -withdrawing properties: *p*-nitrobenzaldehyde, *p*-cyanobenzaldehyde, and 3,4,5-trimethoxy-benzaldehyde (**5a**–**d**). Reactions were performed for 2 h, after which the products were again analyzed by HPLC and LC-MS, and though yields and TTNs were typically low, they were consistent with values reported for several myoglobin variants (Fig. 4 and *SI Appendix*, Figs. S22–S24 and Table S1) (37). Interestingly, C45 did not exhibit discernible diastereoselectivity for carbonyl olefination, possibly indicating that the ylide is released from the protein prior to rearrangement and final product formation. Nevertheless, the ability to catalyze these reactions further illustrates the utility of C45 as a general carbene transferase enzyme.

**Table 3.** C45-catalyzed N-H insertion reactions

Product	Substrate	R	% Yield (single)	% Yield (double)	Single/double	TTN	Reaction time, min	TOF/min^−1^
3a	Piperidine	Et	78.9 ± 11.9	n/a	n/a	789	120	7.99
3b	Piperidine	^*t*^Bu	62.1 ± 8.9	n/a	n/a	621	120	5.17
3c	Piperidine	Bn	88.3 ± 13.1	n/a	n/a	883	120	7.36
4a	*p*-chloroaniline	Et	67.6 ± 0.3	5.21 ± 0.3	12.7:1	676*	120	5.63
4b	*p*-chloroaniline	^*t*^Bu	72.5 ± 0.2	0	0	725	120	6.04
4c	*p*-chloroaniline	Bn	89.4 ± 0.3	0	0	894	120	7.45

Values are ± SDs. n/a, not applicable.

* Indicates that this TTN corresponds solely to the single insertion product.

### C45-Catalyzed Ring Insertion In Vitro and In Vivo.

**Fig. 5.**
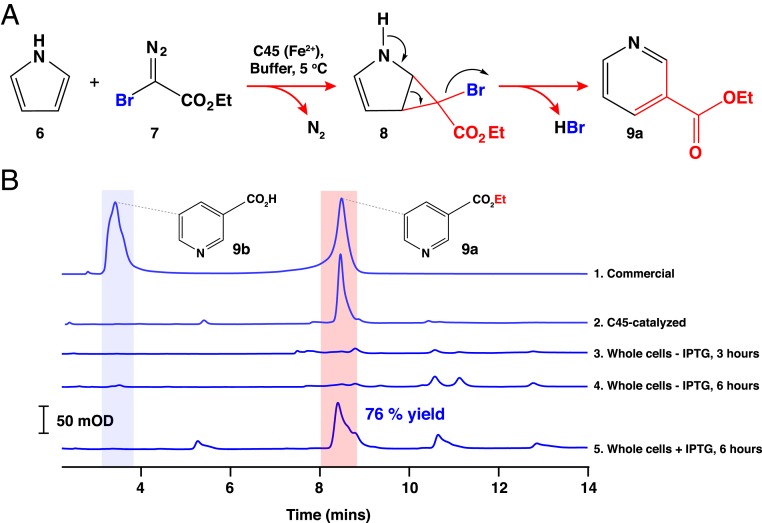
Heteroaromatic ring expansion catalyzed by C45. (*A*) Reaction scheme for the ring-expansion strategy using ethyl 2-bromo-2-diazoacetate, pyrrole, and ferrous C45. Following carbene transfer to the pyrrole, spontaneous rearrangement of the bicyclic ring system leads to elimination of HBr and formation of a 6-membered pyridine ring. (*B*) C18 reversed-phase HPLC traces of the C45-catalyzed ring expansion of pyrrole to ethyl nicotinate. Traces 1 and 2 show the C45-catalyzed ring expansion compared to a partially hydrolyzed commercial standard of ethyl nicotinate. The ring expansion was carried out with 1% catalyst loading (10 μM C45) at the following concentrations of reagents: 10 mM sodium dithionite, 1 mM ethyl 2-bromo-2-diazoacetate, and 10 mM pyrrole in 100 mM KCl, 20 mM CHES (pH 8.6), and 5% EtOH. Traces 3 to 5 show the results of incubating whole cells containing the C45 expression vector and pEC86 harboring the *E. coli* cytochrome *c* maturation apparatus. Traces 3 and 4 represent reactions between whole cells, pyrrole, and ethyl 2-bromo-2-diazoacetate at 3 and 6 h after inoculation and in the absence of the inducer, IPTG. Trace 5 represents the reaction with C45-expressing whole cells, pyrrole, and ethyl 2-bromo-2-diazoacetate. In this case, the cells were grown for 3 h and induced with 1 mM IPTG, and C45 was expressed for a further 3 h prior to use in the whole-cell transformation. Reaction conditions are fully described in *Experimental Details*.

Ring-expansion reactions are exceptionally useful in organic synthesis, as they provide a reliable and facile method for acquiring large, expanded ring systems (53, 54). Though currently underused, the homologous ring expansion of nitrogen-containing heteroaromatics could be of considerable use in the synthesis of pharmaceuticals and natural products. While both the Buchner ring expansion (55) and Ciamician–Dennstedt reaction (56) proceed via a cyclopropane-containing bicyclic system that is subsequently ring-opened, the ring opening during the latter reaction is facilitated by the expulsion of a halogen-leaving group. We therefore hypothesized that with the halogen-substituted carbene precursor **7**, C45 would catalyze the cyclopropanation of the nitrogen-containing heteroaromatic **6** to produce the cyclopropane-containing bicyclic system **8** susceptible to spontaneous ring opening and rearomatization (Fig. 5*A*). Using the same reaction conditions as those described for C45-catalyzed styrene cyclopropanation, we investigated whether C45 could catalyze the ring expansion of pyrrole (**6**) with ethyl-2-bromo-2-diazoacetate (**7**) (57) as the carbene precursor. After 2 h, the reaction was analyzed by using HPLC and LC-MS, confirming the production of the ring-expanded product, ethyl nicotinate (**9a**) at 69.4% yield (Fig. 5*B* and *SI Appendix*, Figs. S25 and S26). Subsequent base-catalyzed hydrolysis of the product yielded the nicotinamide and NAD(P)H precursor niacin (**9b**), raising the possibility of engineering a life-sustaining, artificial biochemical pathway from pyrrole to nicotinamide reliant on the in vivo activity f C45 or a related de novo-designed enzyme. This is an example of an enzyme—natural, engineered, or de novo—that is capable of catalyzing homologous ring expansion reactions via a carbene transfer mechanism.

### A Possible Role for C45 in an Engineered NAD+ Biosynthetic Pathway.

To further explore the possibility of employing C45 in an essential and life-sustaining pathway from pyrrole to the pyridine nucleotides, we examined the ring expansion of pyrrole to ethyl nicotinate in living *E. coli* cells. While the *E. coli* periplasm is known to be sufficiently oxidizing to promote the effective formation of disulfide bonds, its measured redox potential is −165 mV (58), which is sufficiently low to ensure that a large fraction of C45 would be in the active, ferrous state (6). Using an established procedure for carbene transferase activity under such conditions (27–29), we tested the ability of *E. coli* bearing the C45-expression plasmid and pEC86 (harboring the *c*-type cytochrome maturation apparatus) (59) to perform the ring-expansion reaction of pyrrole with ethyl-2-bromo-2-diazoacetate under anaerobic conditions (Fig. 5*B*). Since the maturation apparatus is constitutively expressed and results in the production of several heme-containing membrane proteins (CcmC, CcmE, and CcmF) (60, 61), it was vital to establish whether any intrinsic ring-expansion activity was detectable in the presence of these proteins. Whole cells that had been grown for 3 and 6 h in the absence of the inducer, isopropyl-β-d-1-thiogalactopyranoside (IPTG), had barely detectable ring-expansion activity, as determined by C18-HPLC analysis (Fig. 5*B*). In contrast, cells that were grown for 3 h, induced with IPTG, and grown for a further 3 h displayed significant ethyl nicotinate formation with a total yield of 76% (Fig. 5*B*), indicating that, under these conditions and in vivo, C45 exhibits the catalytic ring-expansion activity. It also indicates that both pyrrole and ethyl-2-bromo-2-diazoacetate are able to cross the outer membrane and access the periplasmically located C45. Interestingly, despite the production of ethyl nicotinate by the cells, we did not observe an increase in the quantity of niacin in the extract, indicating that *E. coli* possibly lacks—or does not produce an appreciable quantity of—an endogenous periplasmic esterase capable of efficiently hydrolyzing the product. We therefore tested a recombinant *Bacillus subtilis* esterase (62) expressed in, and purified from, *E. coli* for ethyl nicotinate hydrolysis activity. Incubation of this esterase with ethyl nicotinate under near-physiological conditions resulted in the production of niacin (**9b**; *SI Appendix*, Fig. S27), thus highlighting another functional part in a potential biosynthetic pathway from pyrrole to the pyridine nucleotides through niacin.

To this end, we speculate that it is possible to make C45 and the *Bacillus* esterase essential to NAD+ biosynthesis. *E. coli* synthesizes NAD+ through 2 pathways that both involve the production of NaMN (nicotinic acid mononucleotide) (*SI Appendix*, Fig. S28), then NaAD (nicotinate adenine dinucleotide) prior to NAD+ formation (63–66): the de novo pathway, using l-aspartate as a starting material to produce quinolate, which is a NaMN precursor (64); or the pyridine ring salvage pathway, where exogenous niacin or nicotinamide is utilized instead as an NaMN precursor, representing the favored pathway when niacin and nicotinamide are abundant in the environment (66). Knocking out a key enzyme in the aspartate pathway—e.g., nicotinate-nucleotide diphosphorylase—will provide an auxotrophic strain of *E. coli* that, when expressing both C45 and a suitable periplasmically directed esterase [using a signal sequence such as malE as for C45 (6)] in nicotinamide and niacin-lacking media, may be capable of converting pyrrole and ethyl-2-bromo-2-diazoacetate to niacin and, ultimately, NAD+, recovering the deleterious phenotype. Currently, production of such an *E. coli* strain is outside the scope of this study, but this strategy highlights the mechanism by which we can tractably and rationally engineer the metabolism of a bacterium to rely on a de novo enzyme to sustain an essential pathway.

## Conclusions

With this work, we have showcased the exceptionally diverse functionality available in this simple, de novo-designed heme protein and demonstrated that the carbene transferase activity intrinsic to the scaffold compares very favorably to that reported for most engineered natural proteins. The formation of the metallocarbenoid intermediate at the C45 heme facilitates not only the high-yielding cyclopropanation of styrene and its derivatives, but also extends to the insertion of carbenes into N–H bonds, the olefination of carbenes, and a description of enzyme-catalyzed ring expansion of a nitrogen heterocycle. This demonstrates that abiological function is intrinsic to these de novo-designed maquettes and that the higher complexity of natural protein frameworks, such as the globins and cytochromes P450, is not necessary to support reactivity of this type. Given the altered product profile exhibited by AP3.2, we have also demonstrated the flexibility of the maquette framework toward substantial mutational change, profoundly affecting the stereoselectivity of the de novo enzyme. This indicates that further yield enhancements and alternative stereoselectivities for these types of reactions are undoubtedly achievable through directed evolution, consistent with the studies reported for the maquette’s natural counterparts (20, 22, 32). Furthermore, there are many beneficial features of C45 that compare favorably against the work described by others with natural enzymes: C45 is expressed at high levels in *E. coli* and is functionally assembled in vivo (6), eliminating the requirement for the in vitro incorporation of abiological metalloporphyrins (32) and facilitating in vivo function (27); C45 is thermally stable and displays excellent tolerance in organic solvents (6), thereby facilitating its use as a homogenous catalyst in aqueous:organic mixtures. In conclusion, this work demonstrates the biocatalytic potential and reactive promiscuity that maquettes possess, and future work will be concerned with improving the substrate scope and catalytic performance of these novel, de novo enzymes. It also reinforces our previous work (6), demonstrating that maquettes and related de novo proteins are much more than just laboratory curiosities or ornaments and are, instead, powerful, green catalysts that can play a valuable role in facilitating challenging organic transformations.

## Experimental Details

Detailed experimental methods are provided in *SI Appendix*.

### General and Molecular Biology, Protein Expression, and Purification.

All chemicals were purchased from either Sigma or Fisher Scientific, and cloned synthetic genes for *Rma*-TDE and Mb(H64V/V68A) were purchased from Eurofins Genomics. Protein expression and purification in *E. coli* T7 Express (NEB) from the pSHT (for periplasmic expression) or pET45b(+) [for cytoplasmic expression of Mb(H64V/V68A)] vectors were carried out as described (6). epPCR libraries of C45, with a mutation rate of 2- to 3-amino-acid mutations, were produced by using the GeneMorph II EZClone Domain Mutagenesis Kit. The mutation rate was determined by randomly selecting and sequencing 10 colonies after transforming the PCR products into *E. coli* T7 Express cells. This mutation rate was achieved with 18.7 ng of target DNA (C45 coding sequence). AP3.2 and APR1 were randomly selected variants from the epPCR C45 library. Each variant was expressed and purified as described for C45 (6), and the purified proteins were used in the cyclopropanation assays.

### Stopped-Flow Spectrophotometry.

Stopped-flow kinetics were conducted by using an SX20 stopped-flow spectrophotometer (Applied Photophysics) housed in an anaerobic glove box under N_2_ ([O_2_] < 5 ppm; Belle Technology). In the initial experiments, a solution containing a known concentration of reduced C45 or AP3.2 [15 μM, 100 mM KCl, and 20 mM 2-(cyclohexylamino)ethane sulfonic acid (CHES), pH 8.6, reduced with a stoichiometric quantity of Na_2_S_2_O_4_] was placed in 1 syringe, and a 1 to 5 mM solution of a carbene precursor (either EDA, ^*t*^BuDA, or BnDA) in ethanol (20 to 100%) was placed in the 2nd syringe. Fifty microliters from each syringe was simultaneously injected into a mixing chamber, and the progression of the reaction was monitored spectroscopically at 5 °C and 25 °C, over the course of 180 to 1,000 s, to examine the metallocarbenoid formation and porphyrin degradation pathway, respectively. The formation of the metallocarbenoid was monitored by following the time-course profiles at 417 nm and 428 to 437 nm, the Soret peak for reduced C45/AP3.2/*Rma*-TDE, and the metallocarbenoid intermediate, respectively. Final concentrations were 7.5 μM ferrous C45/AP3.2/*Rma-*TDE and 0.5 to 2.5 mM EDA/^*t*^BuDA/BnDA (10 to 50% ethanol). The kinetics of the formation of the metallocarbenoid for C45/*Rma-*TDE were determined by using the same conditions outlined above but using varying concentrations of EDA (50 μM to 1.5 mM). The rate constants were calculated at varying substrate concentrations and plotted, and then the data were fitted by using the following equation for reversible formation of an unobserved tetrahedral intermediate, followed by irreversible removal of dinitrogen to the stable metallocarbenoid species:

$$k_{obs}=k_{lim}\left[S\right]/K_{1}+\left[S\right].\text{where}K_{1}=\left(k_{-1}+k_{2}\right)/k_{1}.$$ [1]

Additional experiments using stopped-flow spectrophotometry were conducted to study the degradation of the metallocarbenoid intermediates in the presence of a suitable substrate. A solution containing a known concentration of ferrous C45/AP3.2/*Rma*-TDE (15 μM, 100 mM KCl, and 20 mM CHES, pH 8.6, reduced with Na_2_S_2_O_4_) was placed in 1 syringe, and an 80:20% ethanol:water solution containing 1 mM carbene precursor (either EDA, ^*t*^BuDA, or BnDA) and 6 mM styrene was placed in the 2nd syringe. Fifty microliters from each syringe was simultaneously injected into a mixing chamber, and the progression of the reaction was monitored spectroscopically, at 5 °C and 25 °C, over the course of 180 to 1,000 s to examine carbene transfer activity. The progress of the reaction was monitored at 428 to 433 nm and 417 nm, respectively. Final concentrations were 7.5 μM reduced C45/AP3.2/*Rma*-TDE, 500 μM EDA/^*t*^BuDA/BnDA, and 3 mM styrene (40% ethanol).

### Carbene Transfer Chemistry.

Unless stated otherwise, all assays were conducted under scrubbed nitrogen inside an anaerobic glove box ([O_2_] < 5 ppm; Belle Technology). The assays were conducted inside 1.5-mL screw-top vials sealed with a silicone–septum-containing cap. All assays were conducted in CHES buffer (100 mM KCl and 20 mM CHES, pH 8.6), except for the assays conducted with Mb(H64V,V68A), which were performed in KPi buffer (100 mM potassium phosphate, pH 7).The final reaction volumes for all assays were 400 μL, unless otherwise stated. For a complete description of individual assay conditions and product characterization by chiral HPLC and LC-MS, please refer to the information provided in *SI Appendix*.

### Whole-Cell C45-Catalyzed Ring-Expansion Experiments.

Overnight starter cultures were prepared by adding 100 μL of carbenicillin (50 mg.mL^−1^) and 100 μL of chloramphenicol (50 mg.mL^−1^, C45 only) to 100 mL of Luria broth (LB) before inoculating the medium with a C45-expressing *E. coli* glycerol stock. Starter cultures were then incubated overnight at 37 °C and 180 rpm. Fifty milliliters of the overnight starter culture was then used to inoculate 1 L of LB containing the same concentrations of antibiotics (see above). The 1-L cultures were grown in a shaking incubator at 37 °C and 180 rpm until an OD_600_ between 0.6 and 0.8 was obtained (usually after 3 h), at which point 980 μL was extracted from both vessels, placed inside separate 1.5-mL screw-top vials, and placed on ice. One milliliter of IPTG solution (1 M stock, 1 mM final concentration at induction) was added to specific cultures to induce protein expression; induced and noninduced cultures were left in the incubator for an additional 3 h (37 °C and 180 rpm). After 3 h, 980 μL was extracted from the vessels and transferred to separate 1.5-mL screw-top vials. The samples were then degassed inside an anaerobic glove box (Belle Technology) for 30 min before 10 μL of pyrrole (1 M stock in EtOH) was added, and the vials were sealed and removed from the glove box. The vials were cooled on ice before 25 μL of ethyl 2-bromo-2-diazoacetate (40 mM stock in CH_2_Cl_2_, nitrogen flushed) was added via a gastight needle; the final concentration of the reagents were 1 mM ethyl 2-bromo-2-diazoacetate and 10 mM pyrrole. After the reactions had been left stirring for 2 h, the samples were quenched with 3 M HCl (30 μL) and extracted with 1 mL of ethyl acetate. Analysis was conducted as reported in *SI Appendix*, *Ring Expansion Assays*.

### Supporting Information.

Supporting methods, spectral and kinetic data, HPLC and chiral HPLC chromatograms, LC-MS and MS data, HPLC calibrations, CD spectra and thermal melts, a computational protein model mapped with AP3.2 mutations, and a scheme of NAD biosynthesis are provided in *SI Appendix*.

### Data Availability.

The data that support the findings of this study are available from the corresponding author upon reasonable request.

## Supplementary Material

Supplementary File
