# Supplementary material for: A de novo peroxidase is also a promiscuous yet stereoselective carbene transferase
Source: Proc Natl Acad Sci U S A. 2020 Jan 2;117(3):1419–28. doi: 10.1073/pnas.1915054117 (PMC6983366; doi:10.1073/pnas.1915054117)
Supplement: Supplementary File [file pnas.1915054117.sapp.pdf]

A *de novo* peroxidase is also a promiscuous yet stereoselective carbene transferase

Richard Stenner[1,2]; Jack W. Steventon[1,3]; Annela Seddon[2,4]; J. L. Ross Anderson[1,3*]

[1]School of Biochemistry, University of Bristol, University Walk, Bristol, BS8 1TD, UK.
[2]Bristol Centre for Functional Nanomaterials, HH Wills Physics Laboratory, University of Bristol, Tyndall Avenue, Bristol, BS8 1TL, UK.
[3]BrisSynBio Synthetic Biology Research Centre, Life Sciences Building, University of Bristol, Tyndall Avenue, Bristol BS8 1TQ, UK.
[4]School of Physics, HH Wills Physics Laboratory, University of Bristol, Tyndall Avenue, Bristol, BS8 1TL, UK.

email: ross.anderson@bristol.ac.uk

Supporting Information

www.pnas.org/cgi/doi/10.1073/pnas.1915054117

Supplementary materials and methods.

Protein mass spectrometry for C45

The formation of the C45-metallocarbenoid intermediate was further examined using positive electron-spray-ionization (ESI) mass spectrometry (Waters Xevo G2-XS QTof). A 1 mL solution of 350 µM C45 in CHES buffer (100 mM KCl, 20 mM CHES, pH 8.6), in a 1.5 mL sealed-top vial containing a silicon septum, was flushed under scrubbed nitrogen inside an anaerobic glovebox ([O_2] < 5 ppm; Belle Technology) before being sealed, removed from the glovebox and placed on ice for 10 minutes. The sample was then immediately loaded directly into the mass spectrometer, where an isocratic 100 mM ammonium acetate solution was employed as the mobile phase (0.25 $mL.min^{-1}$). 20 µL injections were employed and the progression of the sample into the chamber was monitored spectroscopically at 280 nm. The mass spectrum contained peaks screened across a m/z range of 1500-2700. The dominant peaks were identified, and the charges of the fragments were calculated using the molecular mass of C45 (15172.5 Da).

A 1 mL solution of 350 µM C45 in CHES buffer (100 mM KCl, 20 mM CHES, pH 8.6), in 1.5 mL sealed-top vials, was then flushed under scrubbed nitrogen inside an anaerobic glovebox ([O_2] < 5 ppm; Belle Technology) followed by the addition of 25 µL of $Na_2S_2O_4$ (400 mM stock in CHES buffer). A separate vial containing the selected diazo compound (EDA, ^{t}BuDA, BnDA, 400 mM stock in EtOH) was deoxygenated alongside the vial containing reduced C45. The vials were sealed, transported out of the glovebox and cooled in an ice bath for 10 minutes. Once cooled, 50 µL of the selected diazo compound was added *via* gastight syringe into the vial containing reduced C45 to initiate the reaction. The sample was kept on ice for 1 minute to allow for the formation of the metallocarbenoid intermediate before being directly loaded onto the mass spectrometer. Final reaction concentrations were 350 µM enzyme, 10 mM sodium dithionite, and 20 mM diazo compound. Identical conditions to the experiment with only C45, mentioned above, were employed for each carbene precursor studied.

Cyclopropanation assays

To 370 µL of a 10 µM C45/AP3.2/*Rma*-TDE /Mb(H64V,V68A) solution was added 10 µL of $Na_2S_2O_4$ (400 mM stock; de-ionized water) and the mixture was left to stir for 1 minute, ensuring complete reduction of C45 from Fe^{3+} to Fe^{2+}. 10 µL of the selected styrene (1.2 M stock in EtOH) was added and the reaction left to mix for 30 seconds. A separate vial containing the selected diazo compound (400 mM stock in EtOH) was deoxygenated alongside the reaction vessel. The vials were sealed, transported out of the glovebox and cooled in an ice bath. Once cooled, 10 µL of the diazo compound was added *via* gastight syringe into the reaction vials to initiate the reaction. Final reaction concentrations were 10 µM enzyme (0.1 mol%), 10 mM sodium dithionite, 10 mM diazo compound, and 30 mM styrene. Once mixed, the reactions were stirred on a roller at room temperature. After 2 hours, the reaction was quenched by the addition of 20 µL of 3 M HCl. The vials were subsequently unscrewed, and 1 mL of ethyl acetate was added to the vial. The solution was transferred to a 1.5 mL microcentrifuge tube, vortexed and centrifuged for 1 minute at 13,500 rpm. The upper organic layer was extracted, dried over $MgSO_4$ if necessary, and subsequently used for analysis. Where base hydrolysis of the resultant ester was necessary, 400 µL of 3 M NaOH was introduced to the organic layer and the mixture was left to stir, at room temperature for 30 minutes (the progress of the ester hydrolysis was monitored using TLC (7:3 ethyl acetate:hexane)). Products were analyzed by chiral-HPLC and LC-MS as described below. The product yields, enantiomeric excesses and total turnover numbers (TTN; concentration of product formed/concentration of enzyme) were

calculated *via* external calibration with commercial ethyl 2-phenylcyclopropane-1-carboxylate and 2-phenylcyclopropane-1-carboxylic acid.

N-H insertion assays

N-H insertion assays were conducted under the same reaction conditions as the cyclopropanation assays, with the following alterations: the alkene starting materials were substituted for the selected amines; the reactions were carried out over 2 hours; after quenching, the products were extracted with 1.25 mL of *n*-hexane (1.25 mL of hexane as required for sufficient extraction of the product into the organic phase [monitored by TLC]); the products were subsequently analyzed by chiral-HPLC and LC-MS as described below. The product yield and total turnover numbers where calculated *via* an external calibration with commercial *n*-phenyglycine ethyl ester and *n*-ethylpiperidine acetate (and including a 1.25 multiplication factor to account for product dilutions).

Carbonyl olefination assays

Carbonyl olefination assays were conducted under the same reaction conditions as the cyclopropanation assays, with the following alterations: alkene substrates were substituted for selected benzaldehyde substrates; 10 mM of PPh_3 was included as an additional reagent in the reaction (10 µL of a 400 mM stock in acetone); the reactions were carried out over 2 hours; after quenching, the products were extracted with 1 mL of dichloromethane; the products were analyzed by C18 reverse-phase HPLC and LC-MS as described below. The product yields, cis/trans ratios and total turnover numbers where calculated *via* an external calibration with commercial ethyl cinnamate and cinnamic acid.

Ring expansion assays

Ring expansion assays were conducted under similar reaction conditions as the cyclopropanation assays described above, but with the following alterations: styrene was substituted for pyrrole (400 mM stock in ethanol); ethyl 2-bromo-2-diazoacetate was used as the carbene precursor (40 mM stock in CH_2Cl_2); the products were extracted with 1 mL of ethyl acetate and subsequently analyzed by C18 reverse-phase HPLC and LC-MS as described below. Final reaction concentrations were 10 µM enzyme (1 mol%), 10 mM sodium dithionite, 1 mM diazo compound, and 10 mM pyrrole. The product yields and total turnover numbers where calculated *via* an external calibration with commercial ethyl nicotinate and niacin.

Product characterization by reverse phase and chiral HPLC

All the reactions performed using C45 and AP3.2 were quantified by High Performance Liquid Chromatography (HPLC). Two separate columns were employed for quantification: a chiral column and a reverse phase C18 column.

A chiral-HPLC column (Astec CHIROBIOTIC® V, 250 x 21 mm, 5 µm) was used to analytically quantify the cyclopropanation and N-H insertion assays, employing an isocratic polar organic mobile phase (100% CH_3CN: 0.1% v/v TFA: 0.1% v/v: Et_3N; 0.1 $mL.min^{-1}$ flow rate and 2 µL injection volume for the cyclopropanation assays; 0.2 $mL.min^{-1}$ flow rate and 5 µL injection volume for the N-H insertion assays). Injection volumes of the N-H insertion assay products were 5 µL to account for the 2.5:1 dilution in the extraction step. All elution traces were monitored spectroscopically at 245, 254 and 280 nm. The chiral-HPLC column allowed the retention times of the starting materials and the reaction products to be determined, and to quantify, where necessary, the enantioselectivity of a given reaction.

A C18 HPLC reverse phase column (Phenomenex, 150 x 15 mm, 5 µm) was used to analytically quantify the ring expansion and carbonyl olefination assays, employing a gradient as the mobile phase for ring expansion assays (70:30% H_2O:CH_3CN to 10:90% H_2O:CH_3CN; 2 $mL.min^{-1}$ flow rate and 20 µL injection volume) and an isocratic mobile phase for the carbonyl olefination assays (100% CH_3CN: 0.1% v/v

TFA: 0.1% v/v: Et_3N; 2 mL.min^{-1} flow rate and 20 µL injection volume). Injection volumes of the sample mixture were 20 µL to account for the 2.5:1 dilution in the extraction step. All elution traces were monitored spectroscopically at 245, 254, 265 and 280 nm. The C18 column allowed the retention times of the starting materials and the reaction products to be determined, and, where necessary, to quantify the diastereoselectivity of a given reaction.

If appreciable product formation could be detected after the initial HPLC experiments, the products were subsequently analyzed using Liquid chromatography-Mass spectrometry (LC-MS) to assist in product identification (described below).

External calibrations of the anticipated reaction products were conducted using HPLC with commercially available standards. The conditions and mobile phases employed in the product analysis for each assay was employed for the calibrations. Each substrate was prepared at multiple concentrations (i.e. 50-500 µM, 1 mM, 2 mM, 5 mM, 7.5 mM, 10 mM, and 20 mM) and the peak height response in the chromatogram was recorded as a function of concentration. A plot of [substrate] vs peak height engendered a straight line which could be used to determine the product yields and TTNs for each assay. Injection volumes for the external calibrations were 2 µL and 8 µL for the chiral-HPLC and C18 columns respectively.

Product characterization by liquid chromatography-mass spectrometry

Liquid chromatography-Mass spectrometry (LC-MS, C8) was employed to identify the products formed in each assay. A C8 reverse column (Grace Vydac, 100 x 21 mm, 5 µm) was used with a 10 minute gradient mobile phase (95:5% H_2O:CH_3CN to 10:90% H_2O:CH_3CN; 0.1% v/v formic acid, 0.25 mL.min^{-1}). Injection volumes of 20 µL were employed and the chromatogram was screened across a wavelength range of 240-300 nm. After eluting from the column, the mixture entered an isocratic solvent chamber where a 1:100 dilution was performed prior to injecting the sample into a positive electron-spray-ionization (ESI) mass spectrometer (Waters Xevo G2-XS QTof). The mass spectrum contained peaks screened across a m/z range of 70-250. Retention times and fragmentation patterns were initially determined using commercial samples of the anticipated products.

Synthesis of external standards

External standards for every reaction (excluding carbonyl olefinations) were synthesised using the following methods:

For cyclopropanation standards: A 100 ml oven-dried round-bottom flask was purged with nitrogen and 7 ml of CH_2Cl_2, $Rh_2(OAc)_4$ (5 mol%) and the appropriate reagent (5 equivalents) were added. A 3 ml solution containing the diazo compound (CH_2Cl_2, 1 equivalent) was added dropwise to the solution while stirring over a 30-minute period. Final concentrations were 250 mM olefin, 50 mM diazo compound and 5% mol catalyst; the final volume was 10 ml. The reaction was left stirring for 1 hour before the solvent was removed under vacuum. The crude mixture was purified by flash column chromatography (9:1 hexanes:diethyl ether) to yield the pure product. The product identify was confirmed *via* ESI-MS and chiral-HPLC. ***Ethyl 2-(4-hydroxyphenyl)cyclopropane-1-carboxylate*:** synthesised following the outlined protocol, colourless oil, % yield (56), LC-MS m/z (% relative intensity): 133.06 (42.48), 207.1 (100). ***Ethyl 2-(4-methoxyphenyl)cyclopropane-1-carboxylate*:** synthesised following the outlined protocol, white needle-like crystals, % yield (90), LC-MS m/z (% relative intensity): 147.07 (74.03), 160.05 (63.11), 175.07 (100). ***Ethyl 2-(4-fluorophenyl)cyclopropane-1-carboxylate*:** synthesised following the outlined protocol, colourless oil, % yield (67), LC-MS m/z (% relative intensity): 135.05 (40.75), 145.04 (45.70), 163.05 (100), 209.09 (1.14). ***Ethyl 2-(4-chlorophenyl)cyclopropane-1-carboxylate***: synthesised following the outlined protocol, colourless oil, % yield (69), LC-MS m/z (% relative intensity): 144.05 (100),

179.02 (51.04), 225.06 (1.47). ***Ethyl 2-(4-(trifluoromethyl)phenyl)cyclopropane-1-carboxylate***: synthesised following the outlined protocol, colourless oil, % yield (61), LC-MS m/z (% relative intensity): 165.01 (100), 183.03 (91.54), 211.06 (57.11), 239.09 (62.33). ***Ethyl 2-(4-(tert-butyl)phenyl)cyclopropane-1*-carboxylate:** synthesised following the outlined protocol, colourless oil, % yield (79), LC-MS m/z (% relative intensity): 127.05 (86.37), 145.06 (100), 168.08 (72.42), 191.10 (5.15), 247.16 (9.87). ***Ethyl 2-(4-cyanophenyl)cyclopropane-1-carboxylate***: synthesised following the outlined protocol, colourless oil, % yield (47), LC-MS m/z (% relative intensity): 131.05 (42.81), 188.07 (100), 216.1 (10.98). ***tert-Butyl 2-phenylcyclopropane-1-carboxylate***: synthesised following the outlined protocol, colourless oil, % yield (62.00), LC-MS m/z (% relative intensity): 127.05 (100), 145.06 (87.13), 168.07 (52.34), 247.16 (16.22). ***Benzyl 2-phenylcyclopropane-1-carboxylate***: synthesised following the outlined protocol, colourless oil, % yield (56.00), LC-MS m/z (% relative intensity): 91.05 (100), 165.05 (44.34), 181.09 (75.77).

For N-H insertion standards: A 100 ml oven-dried round-bottom flask was purged with nitrogen and 7 ml of CH_2Cl_2, $Rh_2(OAc)_4$ (5% mol) and the appropriate reagent (5 equivalents) were added. A 3 ml solution containing the diazo compound (CH_2Cl_2, 1 equivalent) was added dropwise to the solution while stirring over a 30-minute period. Final concentrations were 250 mM olefin, 50 mM diazo compound and 5 mol% catalyst; the final volume was 10 ml. The reaction was left stirring for 4 hours before the solvent was removed under vacuum. The crude mixture was purified by flash column chromatography (8:2 pet ether:ethyl acetate) to yield the pure product. The product identify was confirmed via ESI-MS and chiral-HPLC. ***Ethyl (4-chlorophenyl)glycinate***: synthesised following the outlined protocol, white crystals, % yield (45), LC-MS m/z (% relative intensity): 140.11 (100), 142.09 (39.56), 214.06 (3.41). ***tert-Butyl (4-chlorophenyl)glycinate*:** synthesised following the outlined protocol, yellow oil, % yield (26), LC-MS m/z (% relative intensity): 140.04 (100), 142.04 (31), 226.05 (10.48), 228.05 (3.45). ***Benzyl (4-chlorophenyl)glycinate*:** synthesised following the outlined protocol, yellow oil, % yield (38), LC-MS m/z (% relative intensity): 91.06 (100), 276.01 (14.9), 278.13 (4.3). ***tert-Butyl 2-(piperidin-1-yl)acetate*:** synthesised following the outlined protocol, yellow oil, % yield (47), LC-MS m/z (% relative intensity): 98.10 (100), 144.10 (57.93). ***Benzyl 2-(piperidin-1-yl)acetate***: synthesised following the outlined protocol, yellow oil, % yield (48), LC-MS m/z (% relative intensity): 91.05 (100), 142.08 (17.05), 234.14 (69.87).

Synthesis of ethyl 2-bromo-2-diazoacetate

Ethyl 2-bromo-2-diazoacetate was synthesized as previously reported (57). Briefly, *N*-bromosuccinimide (26 mmol) was added to a solution of EDA (20 mmol) and 1,8-Diazabicyclo(5.4.0)undec-7-ene (28 mmol) in CH_2Cl_2 (5.0 mL) at 0 °C, and the reaction mixture was stirred for 10 minutes. The crude reaction mixture was washed with cold $Na_2S_2O_3$ (aqueous, 3 x 5 mL) and quickly filtered through a silica column (cold CH_2Cl_2). Cold CH_2Cl_2 was added to bring the final volume up to 2.5 mL (~40 mM).

Redox potentiometry

The redox potential of AP3.2 was measured as previously described for C45 (1). Briefly, AP3.2 (50 µM) was loaded into a spectroelectrochemical cell in 100 mM KCl, 50 mM CHES, pH 8.6, 10% glycerol with the following redox mediators: 20 µM benzyl viologen, 20 µM anthroquinone-2-sulfonate, 20 µM phenazine, 25 µM 2-hydroxy-1,4-napthoquinone. Potential was applied to the spectroelectrochemical cell (platinum working and counter electrodes, Ag/AgCl reference electrode) using a Biologic SP-150 potentiostat in both reductive and oxidative directions to confirm equilibration. AP3.2 heme reduction potential was calculated by plotting the fraction of reduced

protein versus the applied potential, and the data were fitted using the following single electron Nernst function:

2. f(x) = (A+B*10^((E_m-x)/59))/(1+10^((E_m-x)/59))

In equation **2**, A and B are y-axis values at 100% oxidized and reduced heme respectively; E_m is the heme reduction potential.

Circular dichroism spectroscopy

Circular Dichroism spectra were collected using a JASCO J-815 CD polarimeter. AP3.2 was loaded into a 1 mm pathlength quartz cell at 0.01 mg.mL^{-1} in mM KCl, 20 mM CHES, pH 8.58, and far-UV CD spectra were recorded at 100 nm/min with a sensitivity of 50 mdeg. Thermal stability of AP3.2 was assessed by monitoring ellipticity at 222 nm while increasing temperature at a ramp rate of 40 °C/hour with 1 °C intervals. All raw data was converted to mean residue ellipticity (MRE) and the thermal denaturation transition midpoint (T_m) was assessed by plotting the second derivatives of a smoothed thermal denaturation trace where the x-axis intercept corresponds to the T_m.

Hydrolysis of ethyl nicotinate by a *Bacillus subtilis* esterase

To a 100 mL round-bottom flask equipped with a small magnetic stirrer bar was added 19.8 mL of CHES buffer (pH 8.6) and 200 µL of commercial ethyl nicotinate (5 M stock in DMSO). The final concentration of ethyl nicotinate was 50 mM. A 100 µL aliquot of the solution was extracted to an Eppendorf tube for HPLC analysis. 2 mg of esterase from *Bacillus subtilis* (SigmaAldrich) was added to the mixture, then the round-bottom flask was closed with a stopper and the solution was left to stir for 1 hour. After 1 hour, a 1 mL aliquot of the solution was extracted into a 1.5 mL Eppendorf tube, and 100 µL of 3 M trichloroacetate was added to precipitate the protein. The mixture was vortexed, centrifuged (30 seconds, 13,000 rpm) and the resulting solution analyzed directly *via* HPLC. The HPLC protocol for the analysis was identical to the procedure outlined in the ''ring expansion assays" section above.

Supplementary references.

1. D. W. Watkins, J. M. X. Jenkins, K. J. Grayson, N. Wood, J. W. Steventon, K. K. Le Vay, M. I. Goodwin, A. S. Mullen, H. J. Bailey, M. P. Crump, F. MacMillan, A. J. Mulholland, G. Cameron, R. B. Sessions, S. Mann, J. L. R. Anderson, Construction and in vivo assembly of a catalytically proficient and hyperthermostable *de novo* enzyme. *Nature Communications* **8**, 358 (2017).

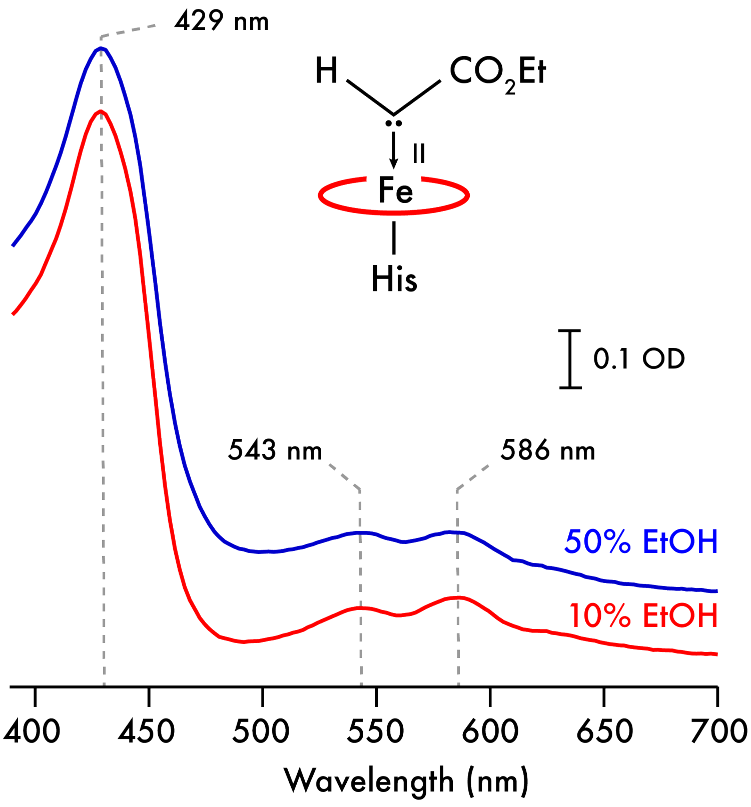

Figure S1. The effect of ethanol on the metallocarbenoid spectra of C45. Electronic spectra were recorded after rapid mixing of ferrous C45 (7.5 µM) with EDA (500 µM) in 10 and 50% ethanol:buffer solutions at 5 °C.

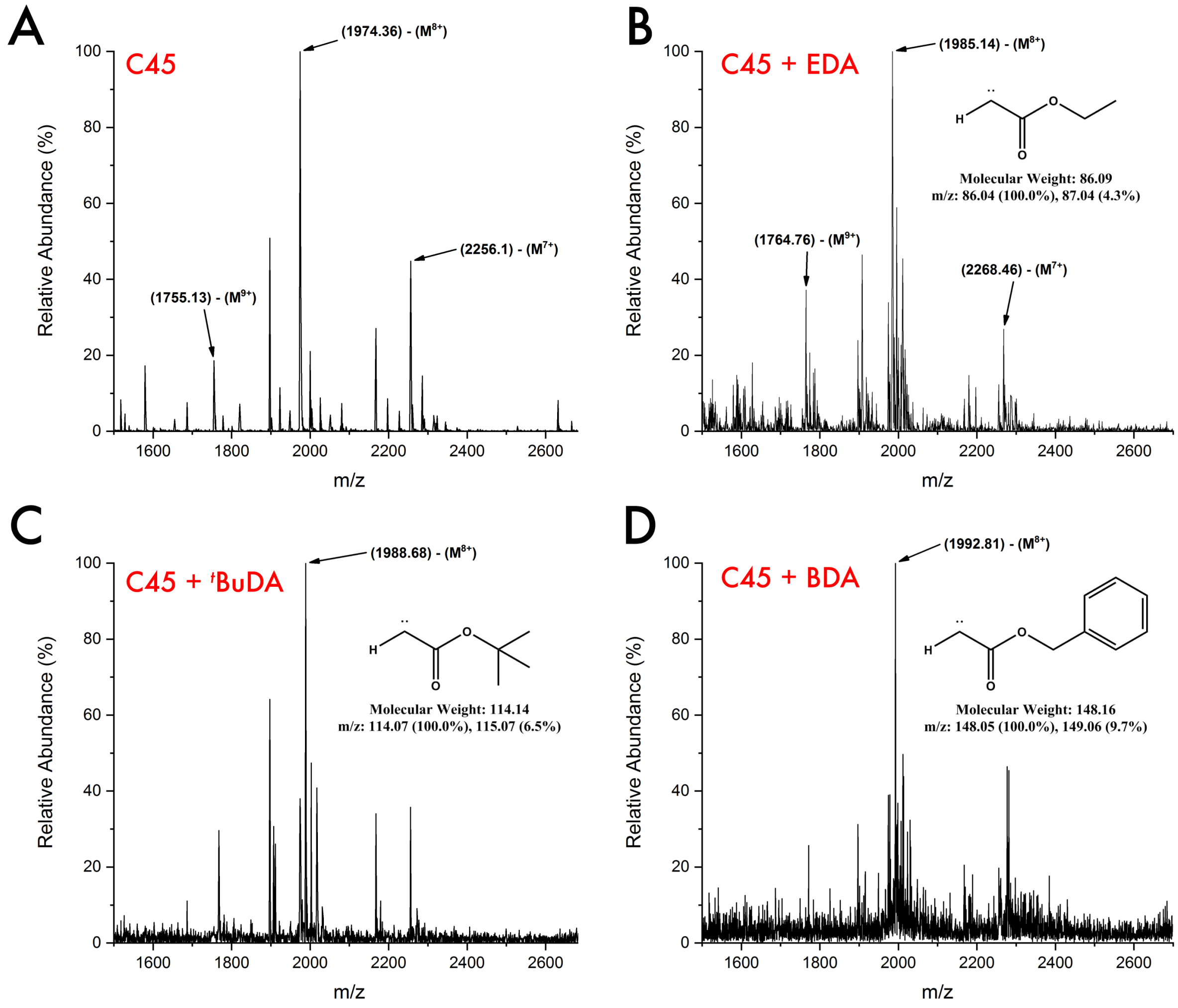

Figure S2. ESI mass spectrometry of C45 metallocarbenoid complexes. A. Mass spectrum of C45 (350 µM) in buffer. The three dominant peaks occur at m/z values of 1755.13, 1974.36, and 2256.1 Da respectively. These fragments correspond to a charged species of (from left to right) 9^+, 8^+, and 7^+, as determined by the atomic mass of C45 (15793.17 Da). **B.** Mass spectrum for C45 after the addition of 50 µl of ethyl diazoacetate (EDA, 20 mM, 5% ethanol). The three dominant peaks in the spectrum have shifted, relative to the peaks exhibited in the C45 spectrum, to 1764.76, 1985.14, and 2268.46 Da for the M^{9+}, M^{8+} and M^{7+} species respectively. The spectrum indicates an average mass increase of 86.52 Da, which corresponds approximately with the predicated molecular weight of an ethyl diazoacetate carbene ($-N_2$) species (86.09 Da). **C.** Mass spectrum for C45 after the addition of 50 µl of *tert*-butyl diazoacetate (^{t}BuDA, 20 mM, 5% ethanol). The dominant peak corresponding to the M^{8+} species has shifted to an m/z value 1988.68, indicating an increase in mass of 114.56 Da, which corresponds with the predicated molecular weight of a *tert*-butyl diazoacetate carbene ($-N_2$) species (114.14 Da). **D.** Mass spectrum for C45 after the addition of 50 µl of benzyl diazoacetate (BnDA, 20 mM, 5% ethanol). The dominant peak corresponding to the M^{8+} species has shifted to an m/z value 1992.81, indicating an increase in mass of 148.08 Da, which corresponds with the predicated molecular weight with of a benzyl diazoacetate carbene ($-N_2$) species (148.05 Da).

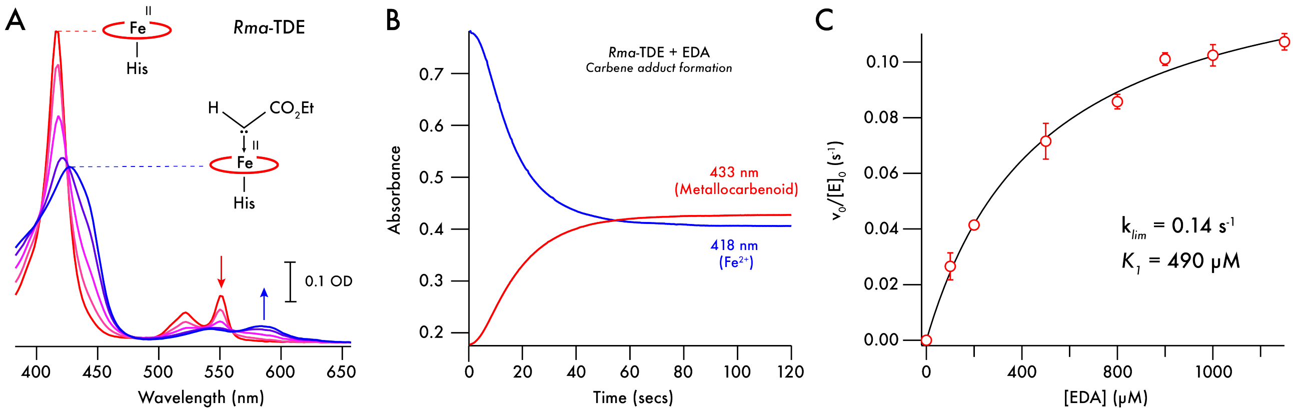

Figure S3. Metallocarbenoid formation and kinetics of *Rma*-TDE with EDA. A. Time course of electronic spectra recorded following rapid mixing of ferrous *Rma*-TDE (7.5 µM, red spectrum) with EDA in 40% EtOH at 5 ºC. The appearance of the metallocarbenoid intermediate (blue spectrum) is concomitant with the disappearance of the ferrous *Rma*-TDE spectrum. Spectra presented were recorded 1, 2, 4, 6, 8, 10, 15, 20, 50 and 120 seconds after mixing. **B.** Metallocarbenoid formation and stability in the absence of styrene substrate. Single wavelength traces represent the time course of ferrous *Rma*-TDE (418 nm, blue; 7.5 µM protein, 100 µM EDA, 40% EtOH, 20 mM CHES, 100 mM KCl, pH 8.6) and metallocarbenoid:*Rma*-TDE adduct (433 nm, red) following rapid mixing of ferrous *Rma*-TDE with 500 µM ethyl diazoacetate at 5 ºC. **C.** EDA-concentration-dependent formation of the *Rma*-TDE metallocarbenoid adduct. Kinetic data were recorded using a stopped-flow spectrophotometer and analyzed as described in the Materials and Methods. The limiting rate constant (k_{lim}) and *pseudo*-Michaelis constant (K_1) for metallocarbenoid formation are 0.14 s^{-1} and 490 µM respectively.

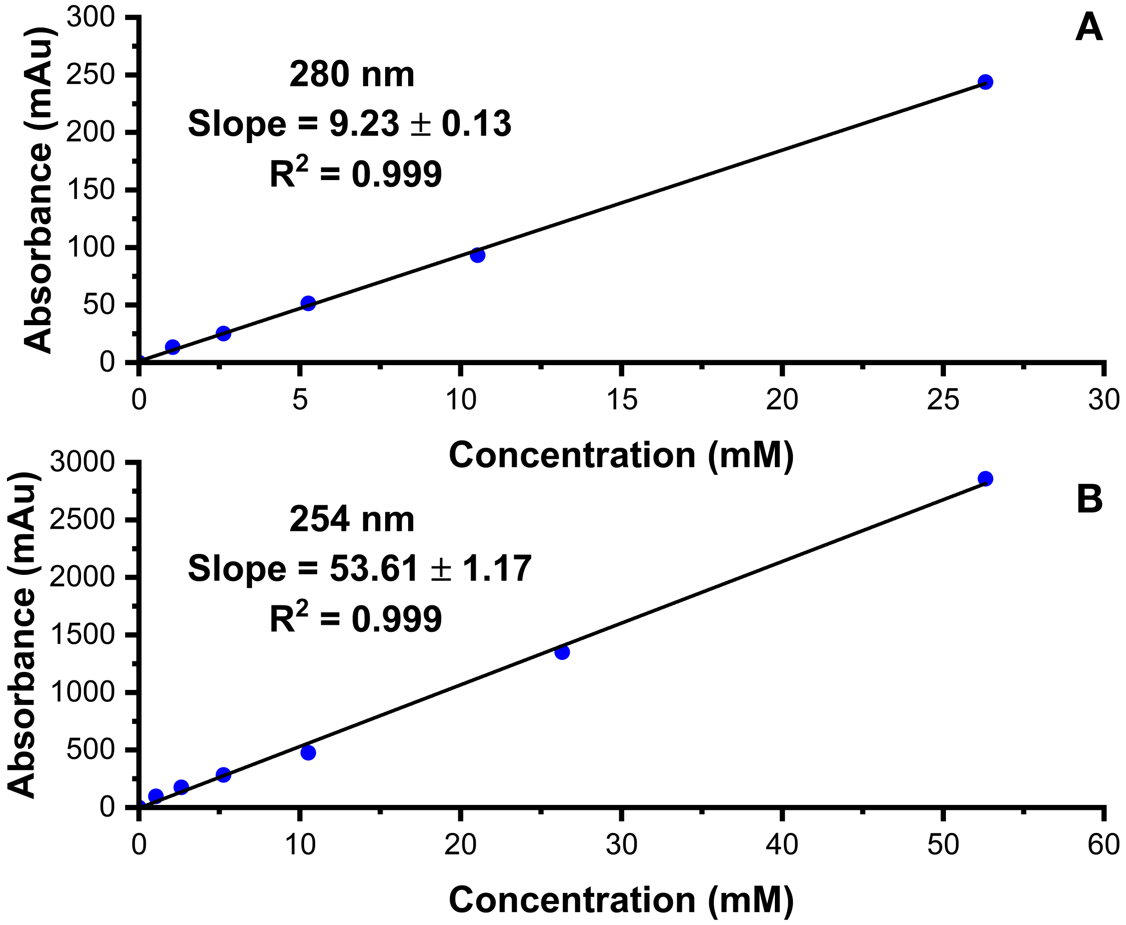

Figure S4. Chiral-HPLC external calibrations for ethyl 2-phenylcyclopropane-1-carboxylate at 280 nm (panel A) and 254 nm (panel B). A polar organic mobile phase (100% MeCN: 0.1% v/v TFA: 0.1% v/v: Et_3N) was employed and injection volumes were 2 µl. These calibrations were carried out on a chiral-HPLC column (Astec CHIROBIOTIC® V, 250 x 21 mm, 5 µm).

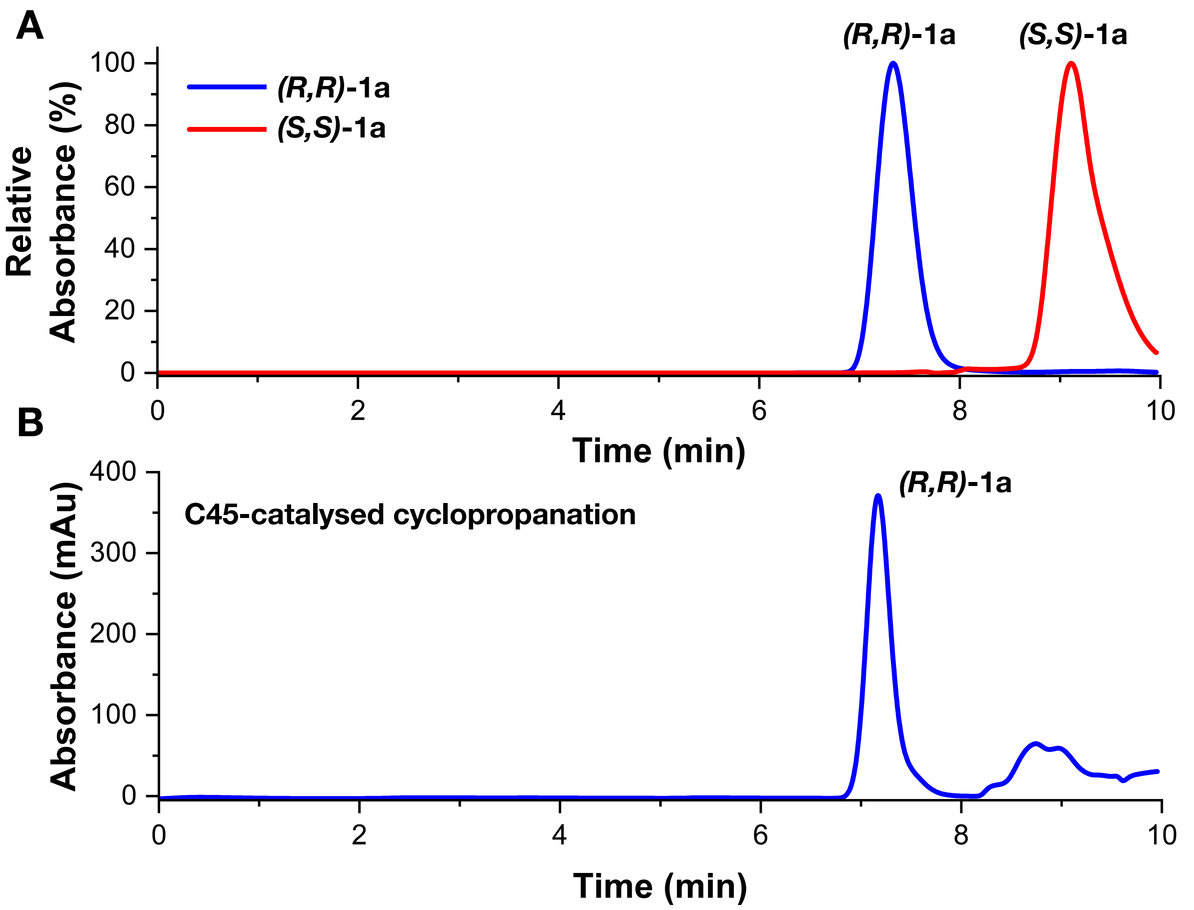

Figure S5. **Chiral-HPLC chromatograms for the cyclopropanation assays.** A polar organic mobile phase (100% MeCN: 0.1% v/v TFA:0.1% v/v: Et_3N) was employed and injection volumes were 2 µl. These data were collected using a chiral-HPLC column (Astec CHIROBIOTIC® V, 250 x 21 mm, 5 µm), and for full experimental details see the *Materials and Methods* section. **A.** Normalised commercial (*R,R*)-ethyl 2-phenylcyclopropane-1-carboxylate (254 nm, EtOH, blue) and (*S,S*)-ethyl 2-phenylcyclopropane-1-carboxylate (254 nm, EtOH, red). **B.** Averaged chromatogram from the C45 (10 µM, 0.1% catalyst loading) catalyzed cyclopropanation assay between styrene (30 mM) and EDA (10 mM) (CHES buffer, pH 8.6, 254 nm). The cyclopropane product from each assay was extracted with 1 mL of ethyl acetate and 400 µl of 3M NaOH prior to loading onto the column. The (*R,R*)-**1a** enantiomer eluted first and was followed by the (*S,S*)-**1a** enantiomer. The relative peak heights for the (*R,R*) and (*S,S*) enantiomers was used to calculate enantiomeric excess values using the equation ([*R,R*]-[*S,S*])/([*R,R*]+[*S,S*]).

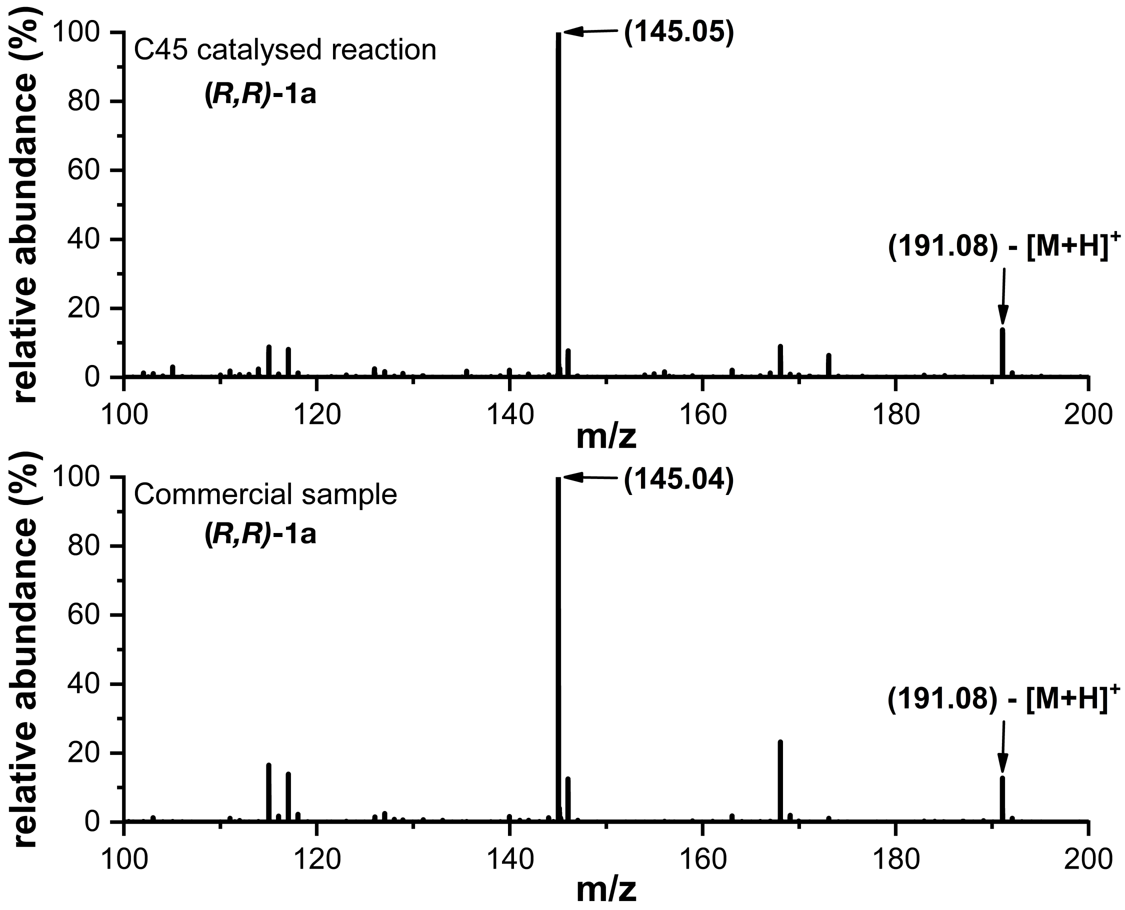

Figure S6. LC-MS spectra of C45 catalyzed cyclopropanation assay products. Commercial ethyl 2-phenylcyclopropane-1-carboxylate (*R,R*)-**1a** (in EtOH) exhibiting the dominant oxonium ion fragment at 145 m/z (top MS) and C45 catalyzed cyclopropanation assay between styrene (30 mM) and EDA (10 mM) (bottom MS). All spectra were recorded in ES+ mode and monitored at 254 and 280 nm. A C8 column was employed for the LC separation with a gradient mobile phase (95:5:0.1% v/v water/MeCN/formate 10:90:0.1% v/v water/MeCN/formate). Assignment of major product peaks in the mass spectra can be found in SI Fig. S9, where R = H.

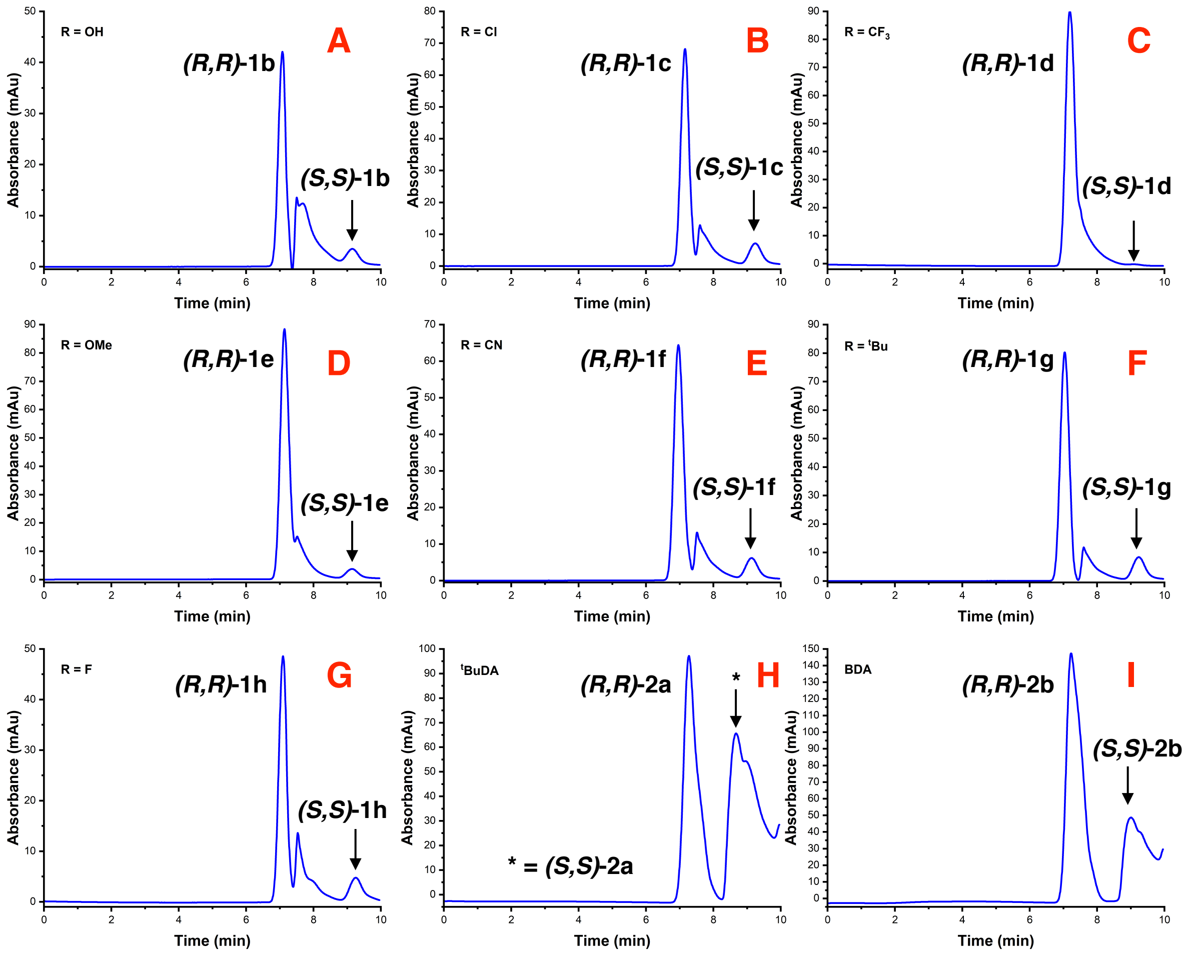

Figure S7. Chiral-HPLC chromatograms for the C45 (10 µM) catalyzed cyclopropanation assays. These data were collected using a chiral-HPLC column (Astec CHIROBIOTIC® V, 250 x 21 mm, 5 µm), and for full experimental details see the *Materials and Methods* section. Crude reaction mixtures for the products of: C45 with *p*-hydroxystyrene (30 mM) and EDA (10 mM), product **1b** (panel **A**); *p*-chlorostyrene (30 mM) and EDA (10 mM), product **1c** (panel **B**); *p*-trifluoromethylstyrene (30mM) and EDA (10 mM), product **1d** (panel **C**); *p*-methoxystyrene (30 mM) and EDA (10 mM), product **1e** (panel **D**); *p*-cyanostyrene (30 mM) and EDA (10 mM), product **1f** (panel **E**); *p*-*tert*-butylstyrene (30 mM) and EDA (10 mM), product **1g** (panel **F**); *p*-fluorostyrene (30 mM) and EDA (10 mM), product **1h** (panel **G**); styrene (30 mM) and *tert*-butyl diazoacetate (10 mM), product **2a** (panel **H**); styrene (30 mM) and benzyl diazoacetate (10 mM), product **2b** (panel **I**). All assays were performed in CHES buffer (pH 8.6) with 10 µM C45 (0.1% catalyst loading). The cyclopropane product from each assay was extracted with 1 mL of ethyl acetate and 400 µl of 3 M NaOH prior to loading onto the column. A polar organic mobile phase (100% MeCN: 0.1% v/v TFA: 0.1% v/v: Et_3N) was employed and injection volumes were 2 µl; all traces were recorded at 280 nm. The (*R,R*)-enantiomer eluted first and was followed by the (*S,S*)-enantiomer. The relative peak heights for the (*R,R*) and (*S,S*) enantiomers was used to calculate enantiomeric excess values using the equation ([*R,R*]-[*S,S*])/([*R,R*]+[*S,S*]).

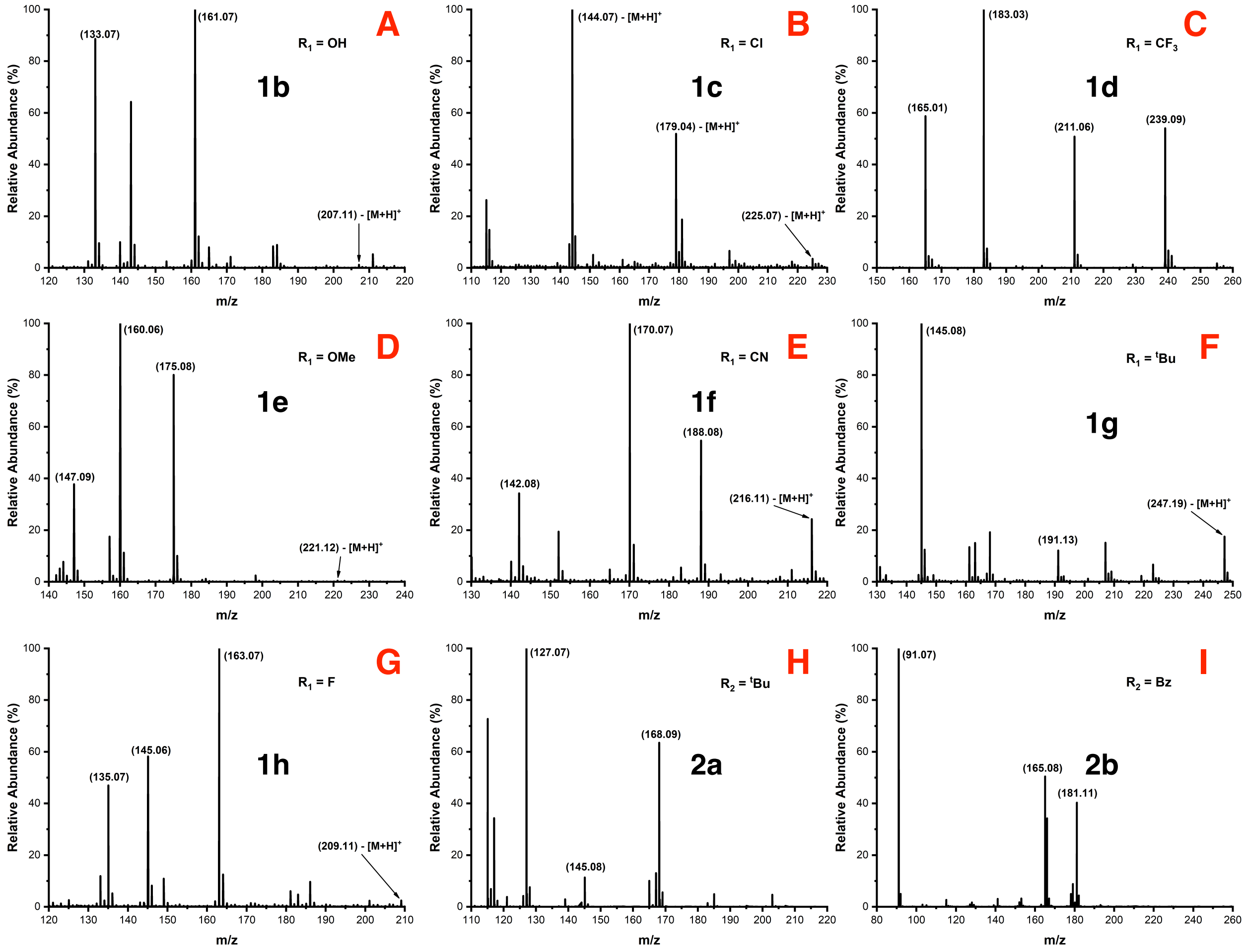

Figure S8. LC-MS spectra and assigned fragments to major product peaks for the C45 (10 μM) catalyzed cyclopropanation assays. LC-MS spectra corresponding to the reaction of: C45 with *p*-hydroxystyrene (30 mM) and EDA (10 mM), product **1b** (panel **A**); *p*-chlorostyrene (30 mM) and EDA (10 mM), product **1c** (panel **B**); *p*-trifluoromethylstyrene (30mM) and EDA (10 mM), product **1d** (panel **C**); *p*-methoxystyrene (30 mM) and EDA (10 mM), product **1e** (panel **D**); *p*-cyanostyrene (30 mM) and EDA (10 mM), product **1f** (panel **E**); *p*-*tert*-butylstyrene (30 mM) and EDA (10 mM), product **1g** (panel **F**); *p*-fluorostyrene (30 mM) and EDA (10 mM), product **1h** (panel **G**); styrene (30 mM) and *tert*-butyl diazoacetate (10 mM), product **2a** (panel **H**); styrene (30 mM) and benzyl diazoacetate (10 mM), product **2b** (panel **I**). All spectra were recorded in ES+ mode and monitored at 254 and 280 nm. A C8 column was employed for the LC separation with a gradient mobile phase (95:5:0.1% v/v water/MeCN/formate 10:90:0.1% v/v water/MeCN/formate). For full experimental details see the *Materials and Methods* section.

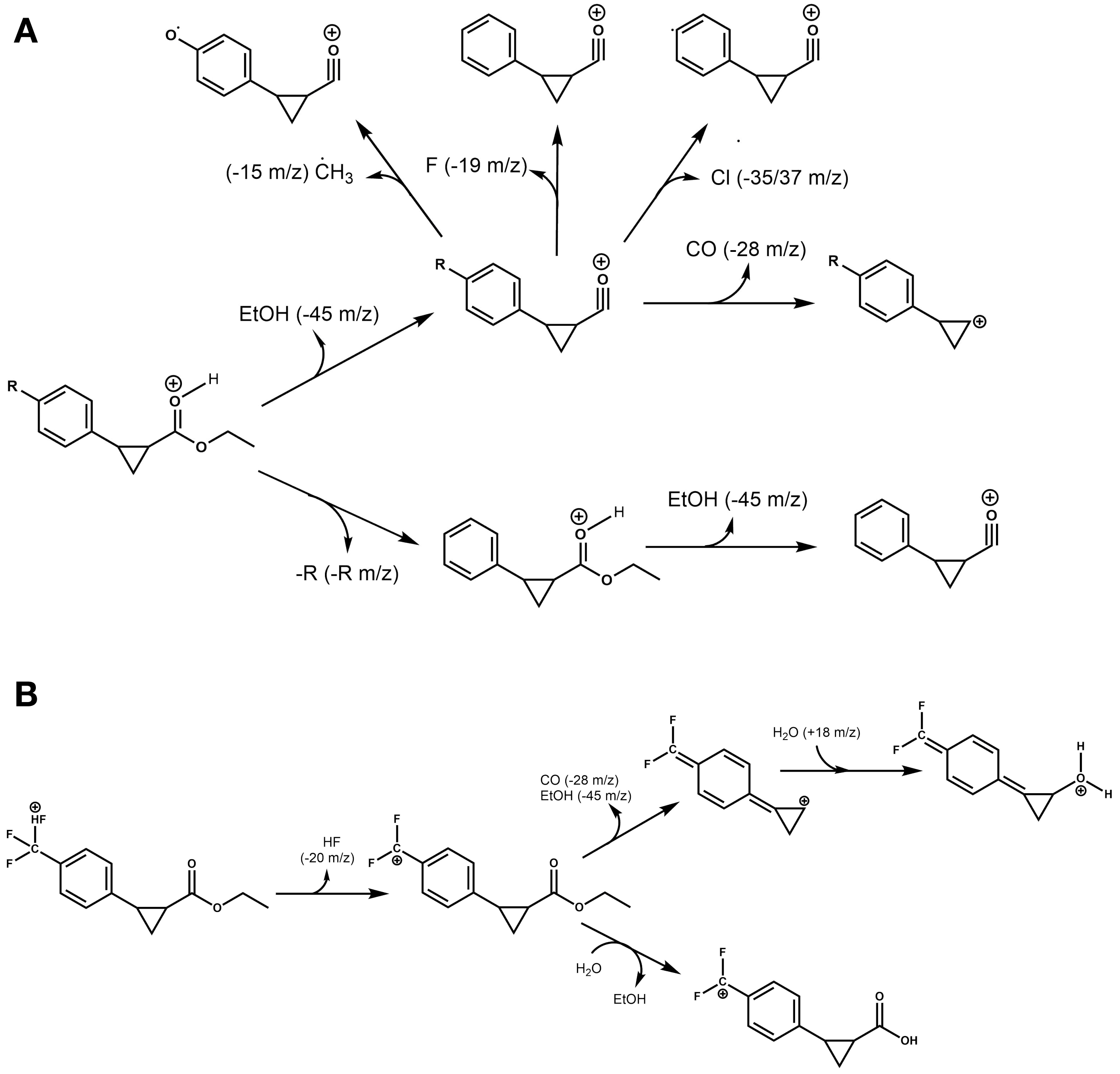

Figure S9. Proposed fragmentation pathways for the products of C45-catalyzed cyclopropanation of *para*-substituted styrenes. A. Fragmentation pathways leading to the observed peaks in the mass spectra. **B.** Fragmentation pathway for the product obtained using *p*-trifluoromethylstyrene as the starting substrate, resulting in the peaks observed in the product mass spectrum.

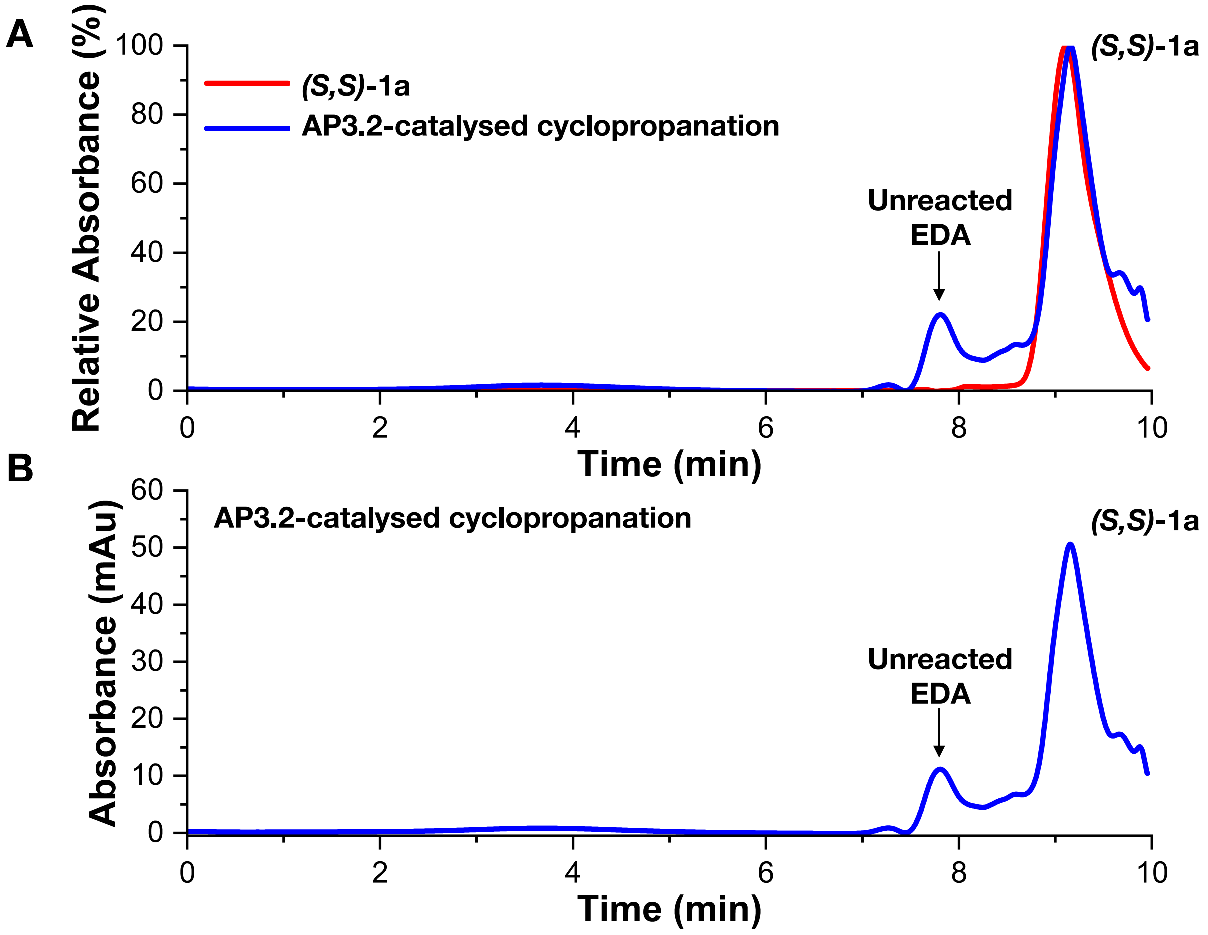

Figure S10. **Chiral-HPLC chromatograms for the AP3.2 catalyzed cyclopropanation assays. A.** normalised AP3.2 (10 µM, 0.1% catalyst loading) catalyzed cyclopropanation assay between styrene (30 mM) and EDA (10 mM) (100 mM KCl, 20 mM CHES, pH 8.6, EtOH, 254 and 280 nm) (blue line) vs normalised commercial (*S,S*)-**1a** (red line). **B.** Averaged AP3.2 (10 µM) catalyzed cyclopropanation assay between styrene (30 mM) and EDA (10 mM) (CHES buffer, pH 8.6, 254 and 280 nm). The cyclopropane product from each assay was extracted with 1 mL of ethyl acetate and 400 µl of 3M NaOH prior to loading onto the column. A polar organic mobile phase (100% MeCN: 0.1% v/v TFA: 0.1% v/v: Et_3N) was employed and injection volumes were 2 µl. The relative peak heights for the (*R,R*) and (*S,S*) enantiomers was used to calculate enantiomeric excess values using the equation ([*R,R*]-[*S,S*])/([*R,R*]+[*S,S*]). These data were collected using a chiral-HPLC column (Astec CHIROBIOTIC® V, 250 x 21 mm, 5 µm), and for full experimental details see the *Materials and Methods* section.

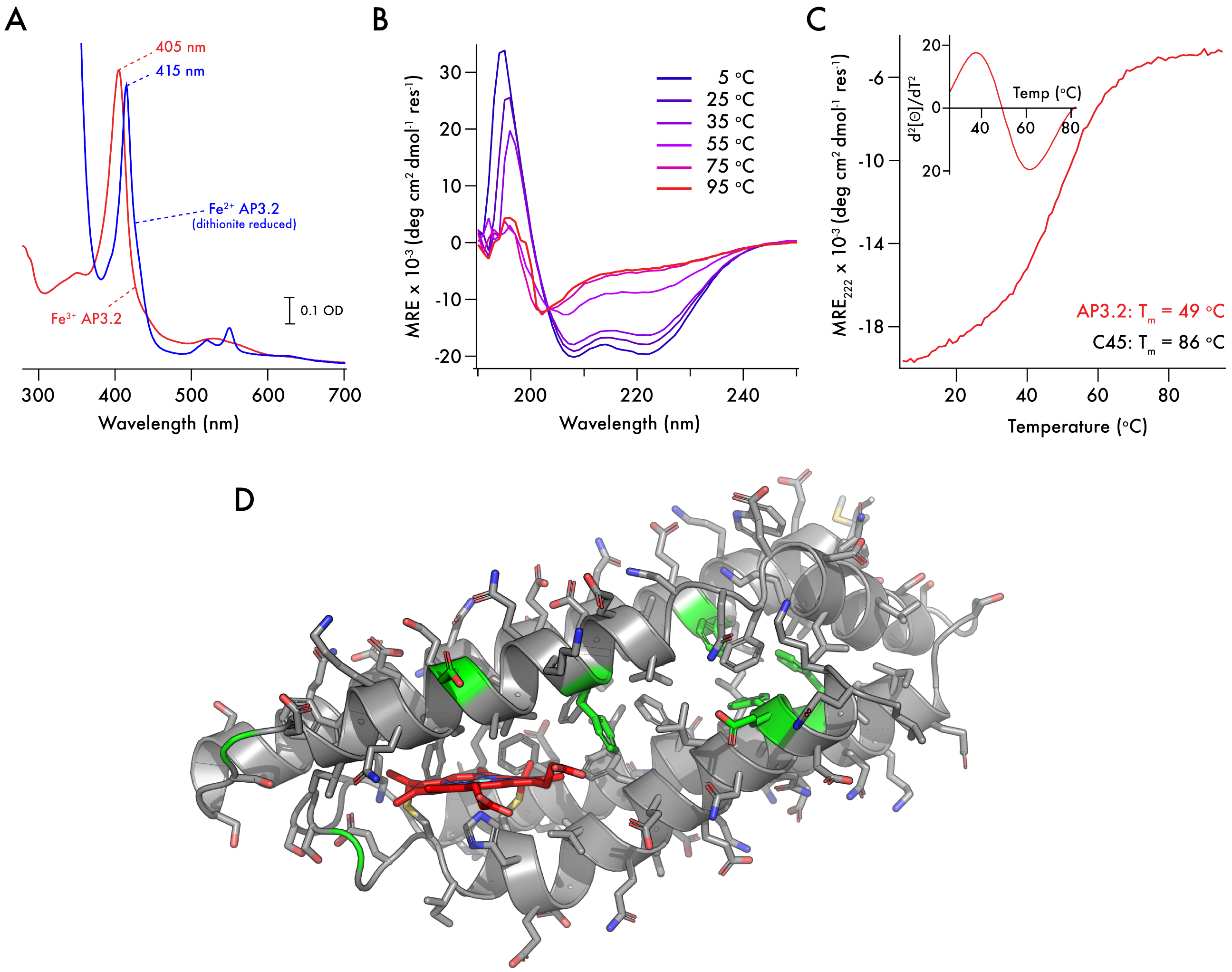

Figure S11. Characterisation of AP3.2. A. UV/visible spectra of ferric (red) and dithionite reduced ferrous AP3.2 (blue). **B.** Far-UV circular dichroism spectra of AP3.2 with varying temperature collected in 100 mM KCl, 20 mM CHES, pH 8.6. **C.** Temperature dependence of the CD signal monitored at 222 nm during thermal denaturation. The inset shows a smoothed second derivative of the thermal melt trace indicating a melting transition (T_m) of 49 °C. **D.** The positions of the eight mutations (F11Y/G39S/D48Y/F53S/F83S/G109A/F132S/E133G) are indicated in green on a computationally-derived model of C45 (6).

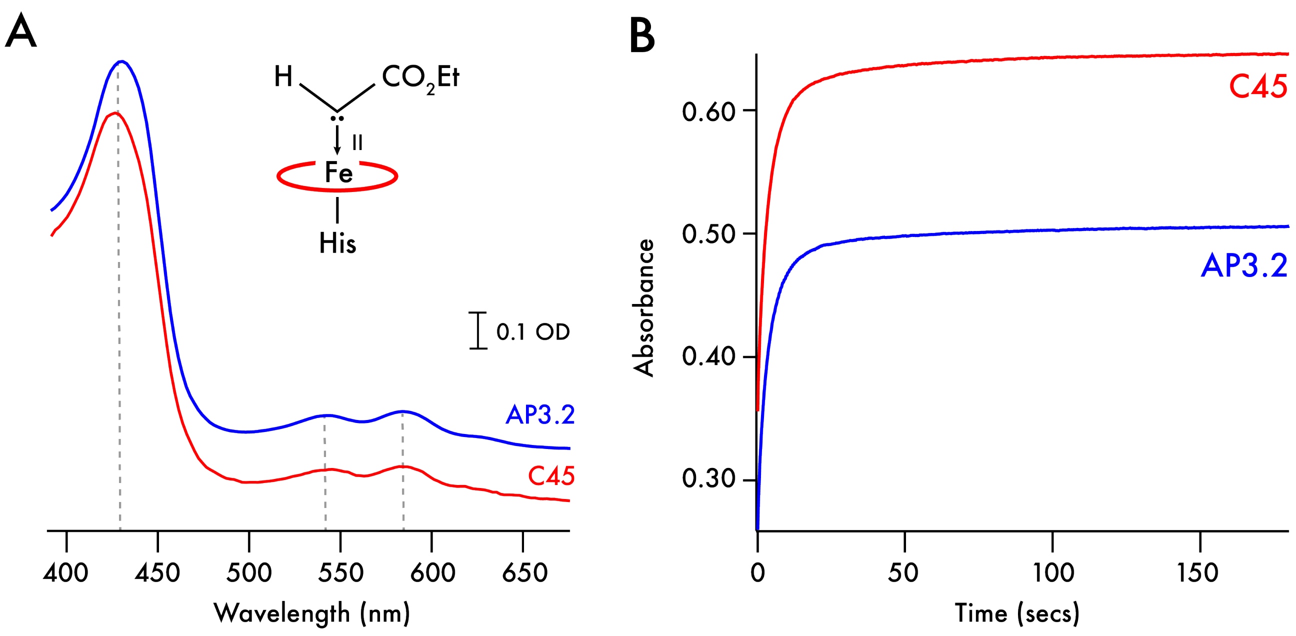

Figure S12. Comparison of metallocarbenoid formation in C45 and AP3.2. A. Visible spectra of the metallocarbenoid intermediates observed for C45 (red) and AP3.2 (blue) after rapid mixing of maquette (7.5 µM final concentration) with 2.5 mM EDA (40% EtOH) at 5 °C in the stopped-flow. **B.** Kinetic traces of metallocarbenoid formation in AP3.2 (blue) and C45 (red) measured at 437 nm after rapid mixing of maquette with 2.5 mM EDA (40% EtOH) at 5 °C in the stopped flow.

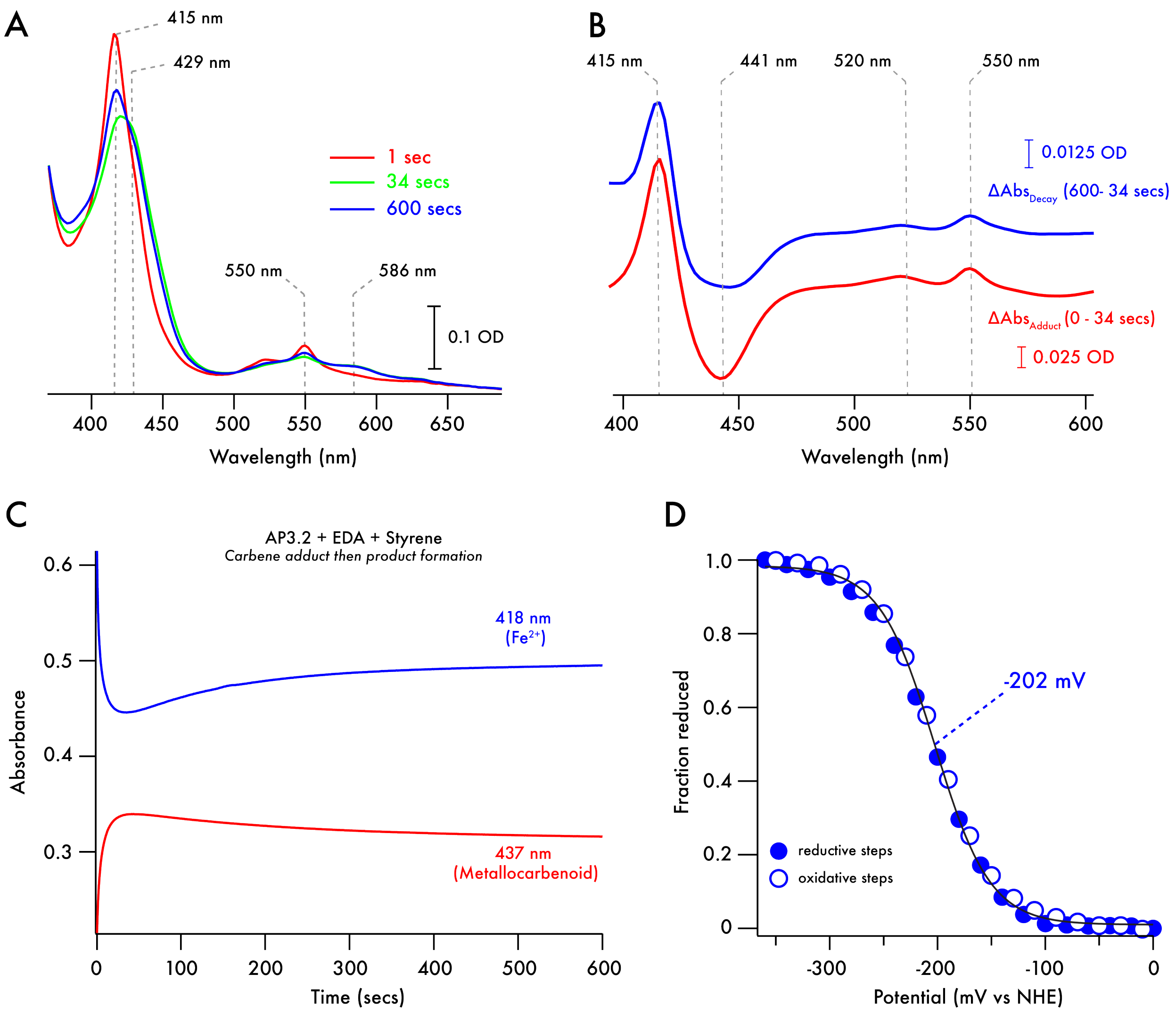

Figure S13. Generation of the AP3.2 metallocarbenoid intermediate in the presence of 3mM styrene. A. Electronic spectra recorded after rapid mixing of ferrous AP3.2 (7.5 µM, red trace) with EDA (500 µM) and styrene (3 mM) at 5 °C (40% EtOH) in the stopped-flow spectrophotometer. The green spectrum taken at 34 seconds post mixing decays towards the ferrous spectrum over the course of 600 seconds (blue trace). **B.** Electronic difference spectra highlighting the spectroscopic changes associated with metallocarbenoid formation and decay in AP3.2. The lower, red trace demonstrates the spectroscopic changes that occur during the formation of the metallocarbenoid, which are similar to those observed during its subsequent decay (upper, blue trace). **C.** Kinetic trace corresponding to the decay and reformation of the ferrous AP3.2 species during turnover. Data was measured at 418 nm following rapid mixing of ferrous AP3.2 with EDA in the stopped flow spectrophotometer at 5 °C. **D.** Redox potentiometry of AP3.2. Spectroelectrochemical data were collected in 100 mM KCl, 50 mM CHES, 10% glycerol, pH 8.6, with redox mediators as described in the methods. UV visible spectra were recorded during stepwise reduction and oxidation of the spectroelectrochemical cell. AP3.2 absorbance at 418 nm was converted to represent the fraction of reduced protein and plotted vs applied potential. Filled circles indicate data collected during the reductive steps, open circles indicate data collected during oxidative steps. Data were fitted to a single electron Nernst model.

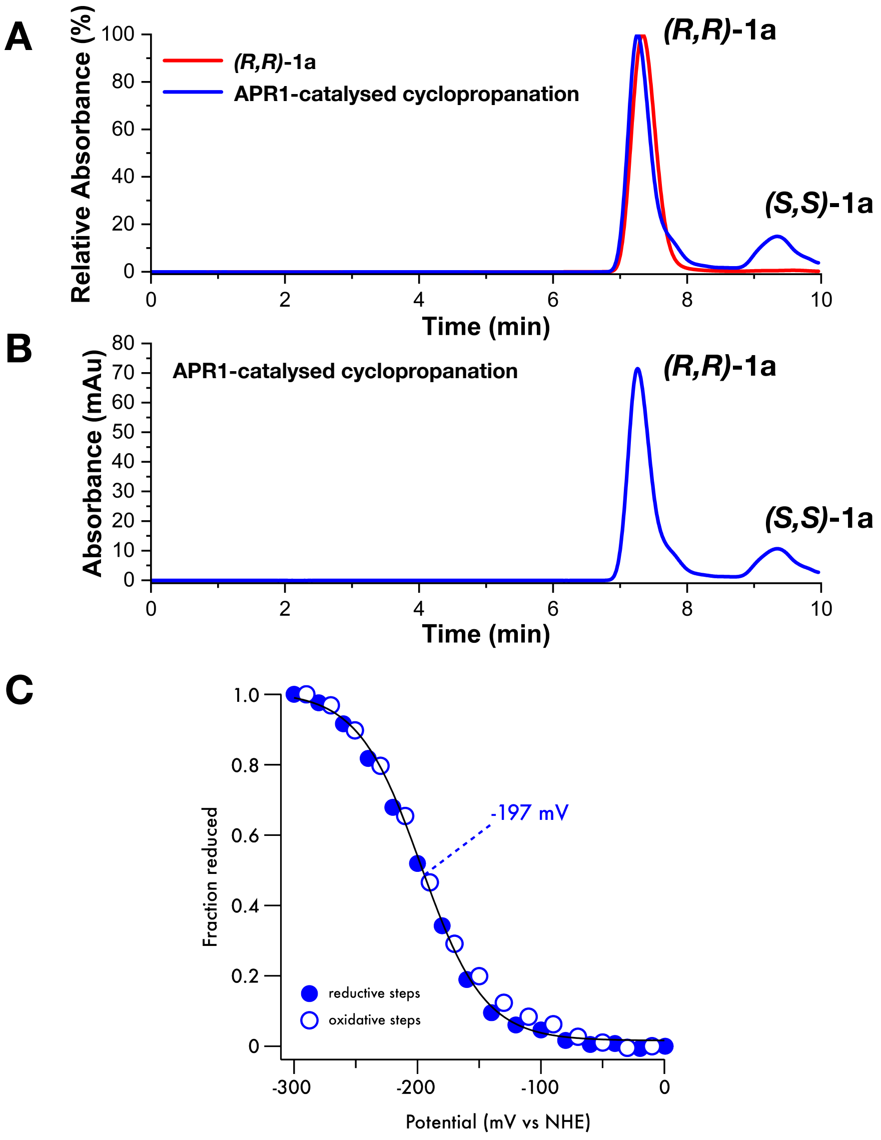

Figure S14. **Chiral-HPLC chromatograms for the APR1 catalyzed cyclopropanation assays. A.** Normalised APR1 (10 µM, 0.1% catalyst loading) catalyzed cyclopropanation assay between styrene (30 mM) and EDA (10 mM) (100 mM KCl, 20 mM CHES, pH 8.6, EtOH, 254 and 280 nm) (blue line) vs normalised commercial (*R,R*)-ethyl 2-phenylcyclopropane-1-carboxylate (red line). **B.** Average APR1 (10 µM) catalyzed cyclopropanation assay between styrene (30 mM) and EDA (10 mM) (CHES buffer, pH 8.6, 254 and 280 nm). The cyclopropane product from each assay was extracted with 1 mL of ethyl acetate and 400 µl of 3M NaOH prior to loading onto the column. A polar organic mobile phase (100% MeCN: 0.1% v/v TFA:0.1% v/v: Et_3N) was employed and injection volumes were 2 µl. The relative peak heights for the (*R,R*) and (*S,S*) enantiomers was used to calculate enantiomeric excess values using the equation ([*R,R*]-[*S,S*])/([*R,R*]+[*S,S*]). **C.** Redox potentiometry of APR1. Spectroelectrochemical data were collected in 100 mM KCl, 50 mM CHES, 10% glycerol, pH 8.6, with redox mediators as described in the methods. UV visible spectra were recorded during stepwise reduction and oxidation of the spectroelectrochemical cell. APR1 absorbance at 418 nm was converted to represent the fraction of reduced protein and plotted vs applied potential. Filled circles indicate data collected during the reductive steps, open circles indicate data collected during oxidative steps. Data were fitted to a single electron Nernst model.

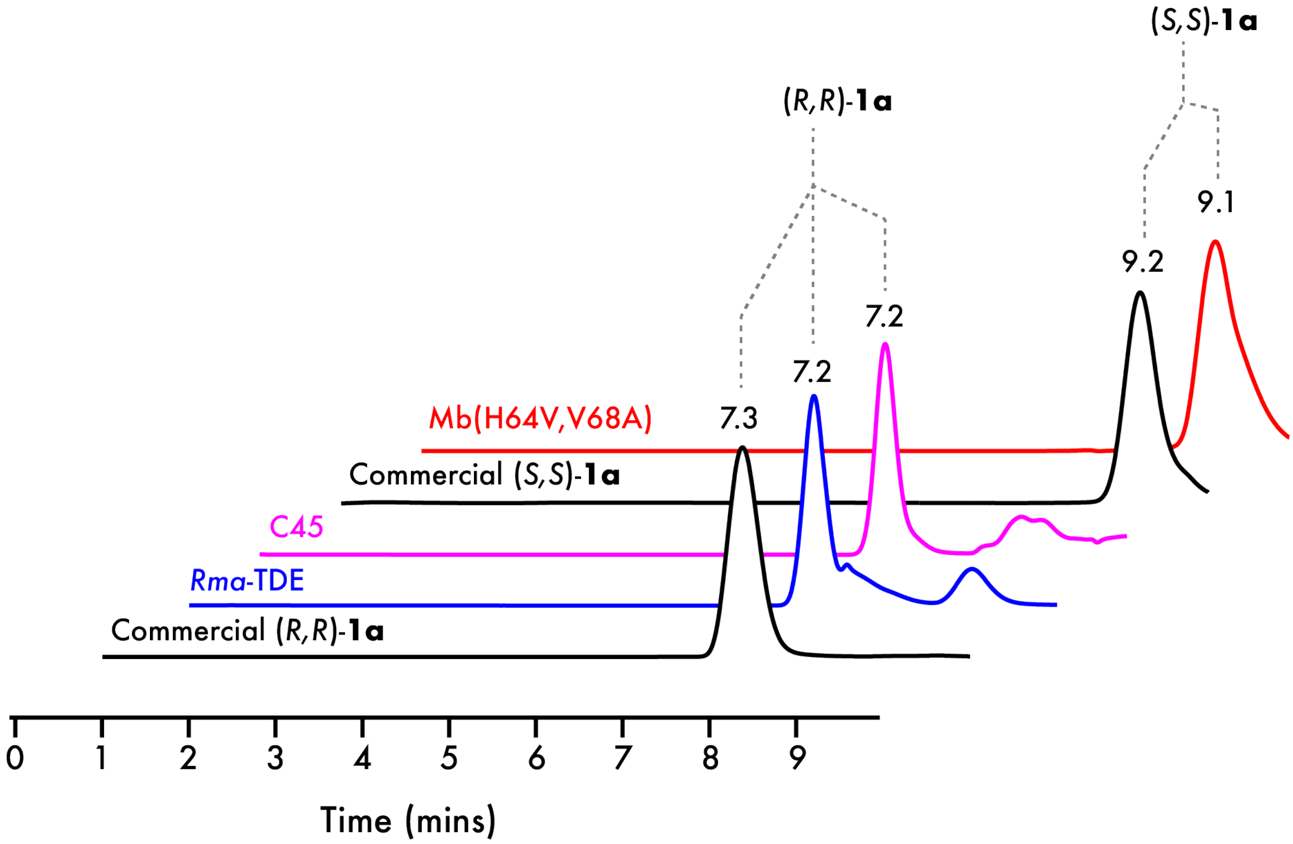

Figure S15. Chiral-HPLC chromatograms of commercial, C45, Mb(H64V,V68A) and *Rma*-TDE catalyzed cyclopropanation assay products. Commercial (*R,R*)-ethyl 2-phenylcyclopropane-1-carboxylate (in EtOH) and commercial (*S,S*)-ethyl 2-phenylcyclopropane-1-carboxylate (in EtOH) are represented by the labelled black traces. Also presented are chromatograms from *Rma*-TDE catalyzed cyclopropanation assay between styrene (30 mM) and EDA (10 mM) (blue trace), the C45-catalyzed cyclopropanation assay between styrene (30 mM) and EDA (10 mM) (magenta trace), and the Mb(H64V,V68A) catalyzed cyclopropanation assay between styrene (30 mM) and EDA (10 mM) (red trace). All assays were conducted in CHES buffer (100 mM KCl, 20 mM CHES, pH 8.6, 5% EtOH) and all enzyme-catalyzed reactions contained 10 µM protein (0.1% catalyst loading). The cyclopropane product from each assay was extracted with 1 mL of ethyl acetate and 400 µl of 3M NaOH prior to loading onto the column. A polar organic mobile phase (100% MeCN: 0.1% v/v TFA:0.1% v/v: Et_3N) was employed and injection volumes were 2 µl. All samples were recorded at 254 and 280 nm. The relative peak heights for the (*R,R*) and (*S,S*) enantiomers was used to calculate enantiomeric excess values using the equation ([*R,R*]-[*S,S*])/([*R,R*]+[*S,S*]). These data were collected using a chiral-HPLC column (Astec CHIROBIOTIC® V, 250 x 21 mm, 5 µm), and for full experimental details see the *Materials and Methods* section.

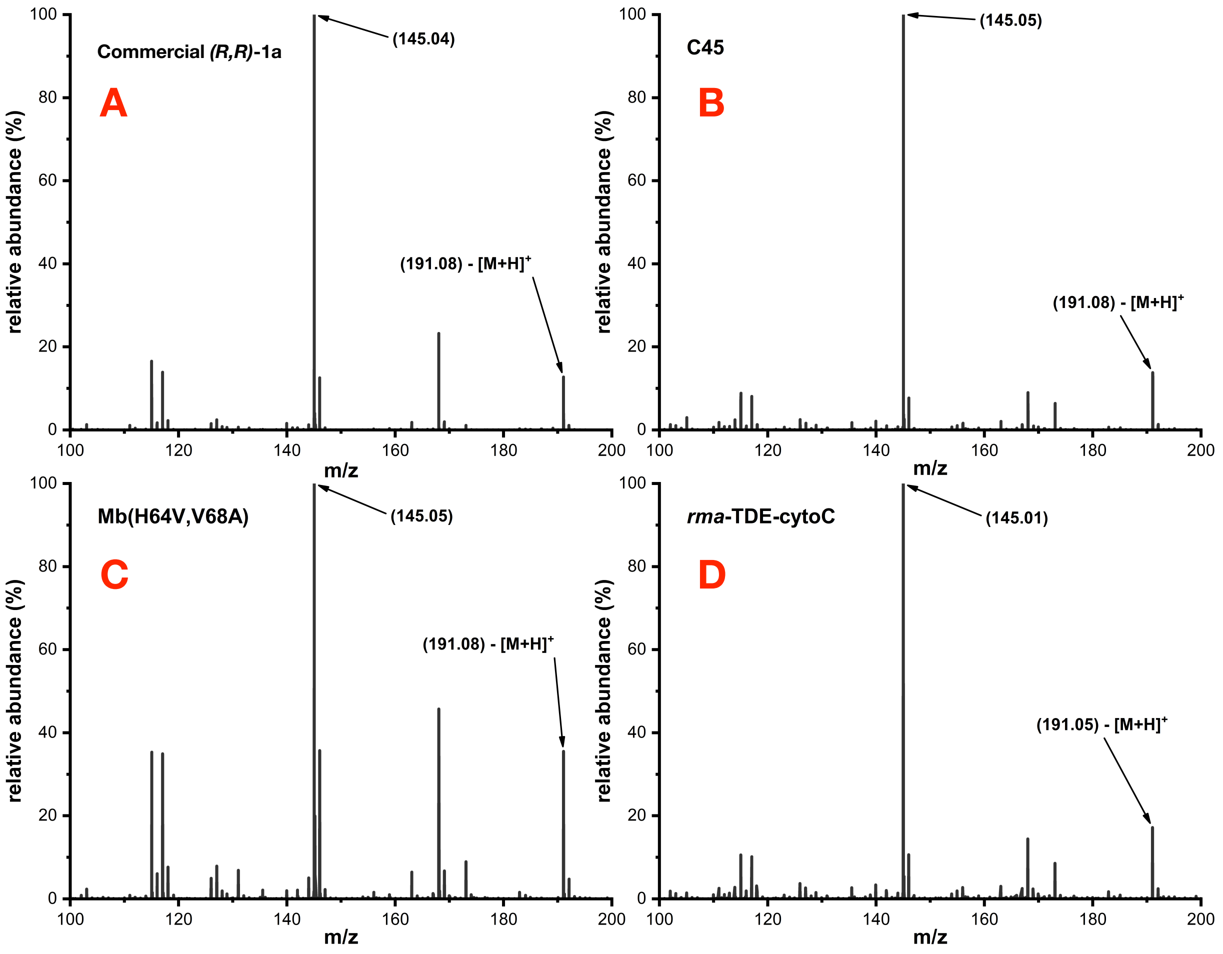

Figure S16. LC-MS spectra of commercial, C45, Mb(H64V,V68A) and *rma*-TDE-cytoC catalyzed cyclopropanation assay products. A. Commercial ethyl 2-phenylcyclopropane-1-carboxylate, *(R,R)*-**1a** (in EtOH), exhibiting the dominant oxonium ion fragment at 145 m/z. **B.** C45-catalyzed cyclopropanation assay between styrene (30 mM) and EDA (10 mM). **C.** Mb(H64V,V68A)-catalyzed cyclopropanation assay between styrene (30 mM) and EDA (10 mM). **D.** *Rma*-TDE-catalyzed cyclopropanation assay between styrene (30 mM) and EDA (10 mM). All spectra were recorded in ES+ mode and monitored at 254 and 280 nm. A C8 column was employed for the LC separation with a gradient mobile phase (95:5:0.1% v/v water/MeCN/formate 10:90:0.1% v/v water/MeCN/formate). Assignment of major product peaks in the mass spectra (bottom).

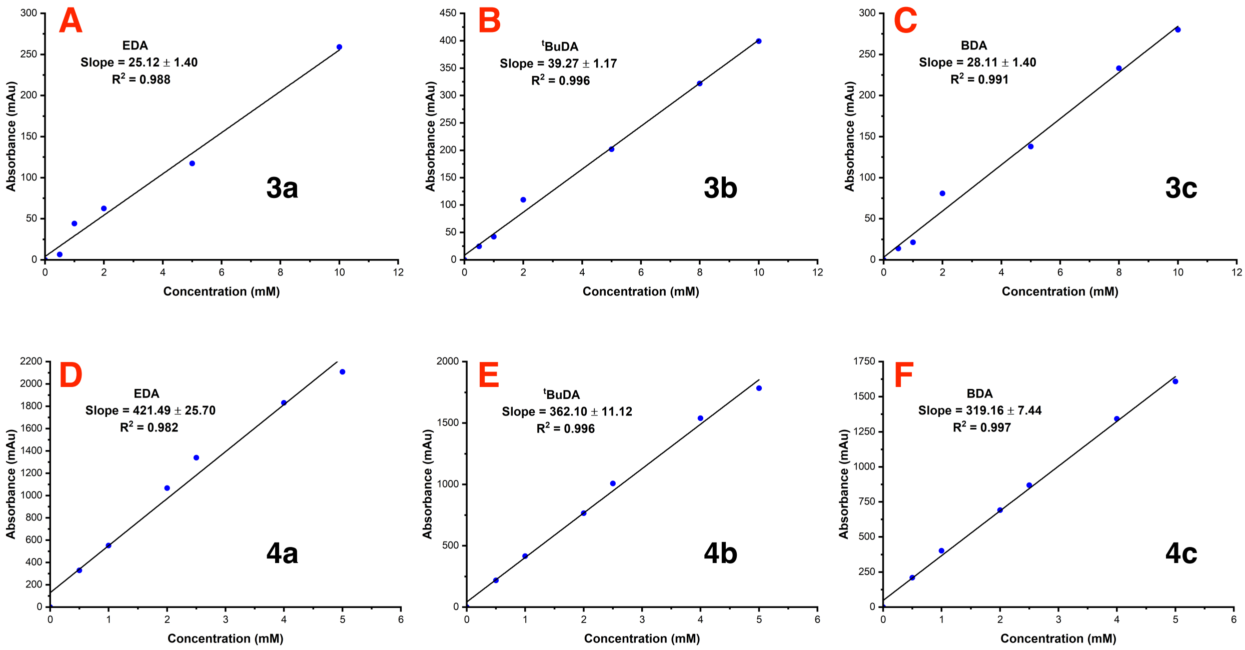

Figure S17. Chiral-HPLC external calibrations for C45-catalyzed N-H insertion reactions. Chiral-HPLC calibrations for synthesised standards: ethyl-1-piperidineacetate, product **3a** (panel A); *tert*-butyl 2-(piperidin-1-yl)acetate, product **3b** (panel B); benzyl 2-(piperidin-1-yl)acetate, product **3c** (panel C); ethyl (4-chlorophenyl)glycinate, product **4a** (panel D); *tert*-butyl (4-chlorophenyl)glycinate, product **4b** (panel E); benzyl (4-chlorophenyl)glycinate, product **4c** (panel F). All calibrations were monitored at 254 nm. A polar organic mobile phase (100% MeCN: 0.1% v/v TFA: 0.1% v/v: Et_3N) was employed and injection volumes were 2 µl. All calibrations were carried out on a chiral-HPLC column (Astec CHIROBIOTIC® V, 250 x 21 mm, 5 µm).

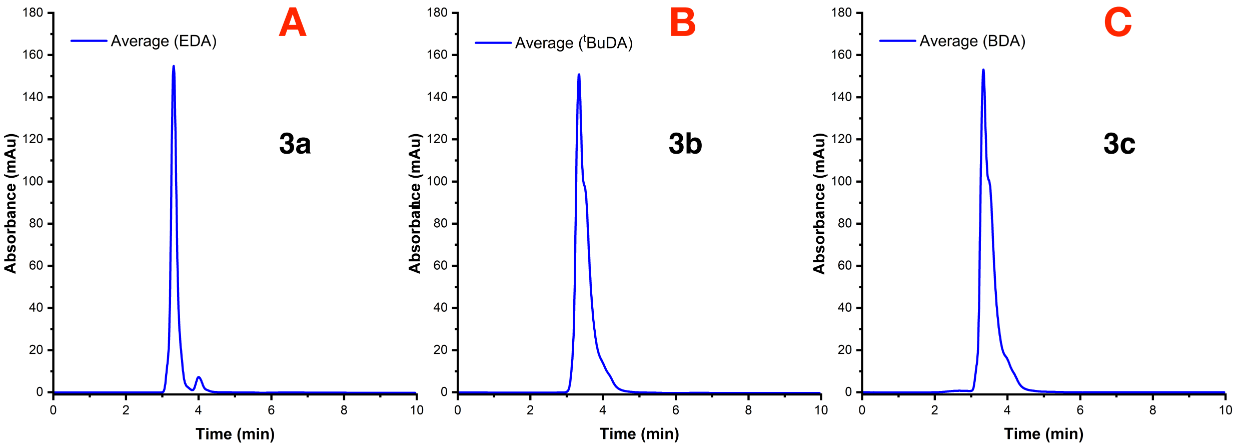

Figure S18. **Chiral-HPLC chromatograms for the C45-catalyzed N-H insertions reactions between piperidine and substituted diazo compounds.** Crude reaction mixtures for the products of the C45-catalyzed reaction between: piperidine (30 mM) and EDA (10 mM), product **3a** (panel A); piperidine (30 mM) and *ᵗ*BuDA (10 mM), product **3b** (panel B); piperidine (30 mM) and BnDA (10 mM), product **3c** (panel C). All reactions were performed under identical conditions (100 mM KCl, 20 mM CHES, pH 8.6, 5% EtOH) with 10 µM C45 (0.1% catalyst loading). The N-H insertion product from each assay was quenched with 3M HCl and extracted with 1.25 mL of hexane prior to loading onto the column. A polar organic mobile phase (100% MeCN: 0.1% v/v TFA:0.1% v/v: Et_3N) was employed and injection volumes were 2 µl. Chromatograms were recorded at 254 nm.These data were collected using a chiral-HPLC column (Astec CHIROBIOTIC®V, 250 x 21 mm, 5 µm), and for full experimental details see the *Materials and Methods* section.

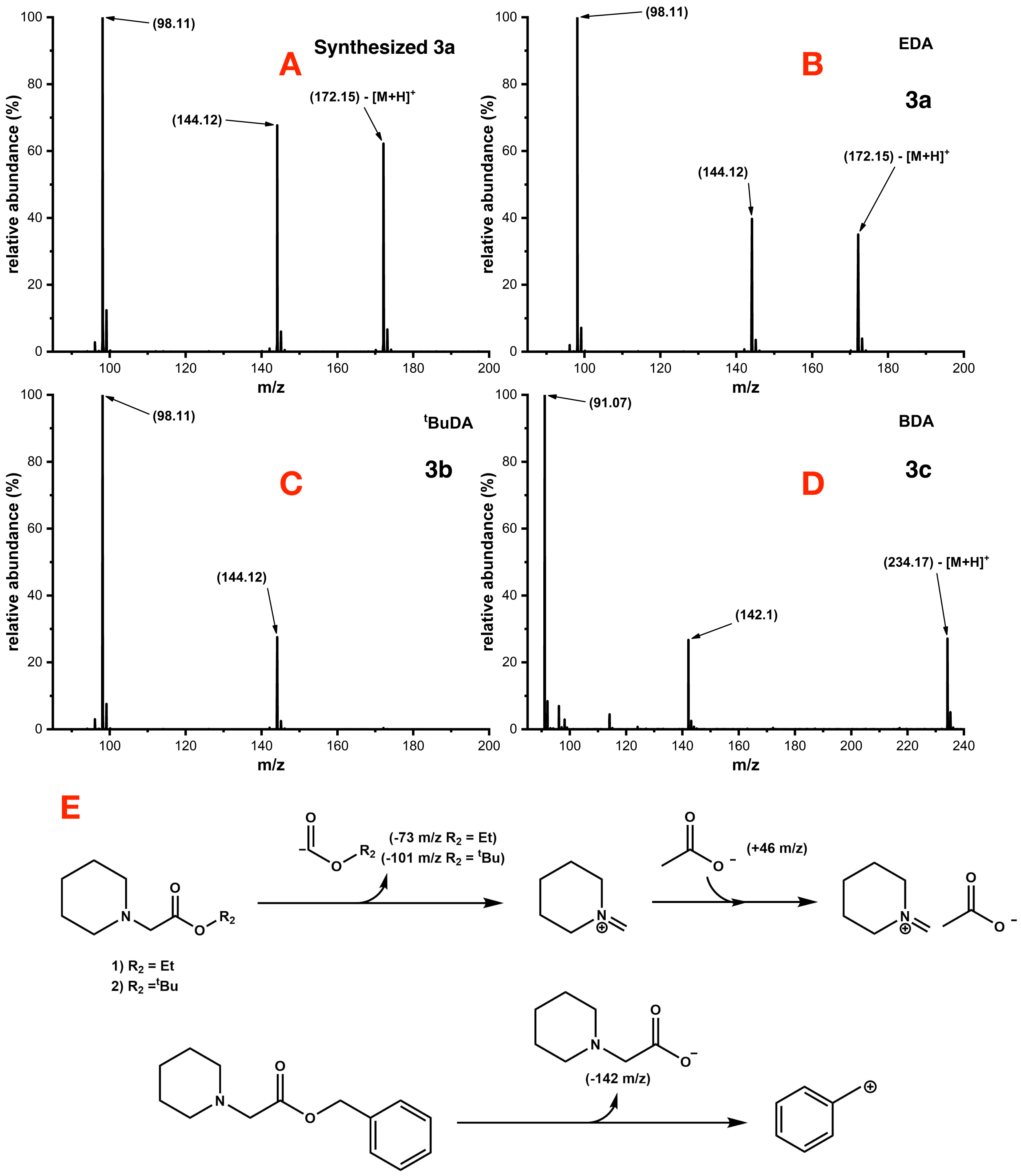

Figure S19. **LC-MS spectra for the C45-catalyzed N-H insertions reactions between piperidine and various diazo compounds. A.** A synthesized sample of ethyl-1-piperidineacetate (in EtOH). **B.** C45-catalyzed N-H insertion assay between piperidine (30 mM) and EDA (10 mM), product **3a**. **C.** C45-catalyzed N-H insertion assay between piperidine (30 mM) and ^{t}BuDA (10 mM), product **3b**. **D.** C45-catalyzed N-H insertion assay between piperidine (30 mM) and BnDA (10 mM), product **3c**. All spectra were recorded in ES+ mode and monitored at 254 and 280 nm. A C8 column was employed for the LC separation with a gradient mobile phase (95:5:0.1% v/v water/MeCN/formate 10:90:0.1% v/v water/MeCN/formate). **E.** Assignment of major product peaks in the mass spectra. Where the $[M+H]^+$ ion is not observed, comparison of MS fragmentation patterns and HPLC elution profiles with synthesised, characterised standards are used to identify product.

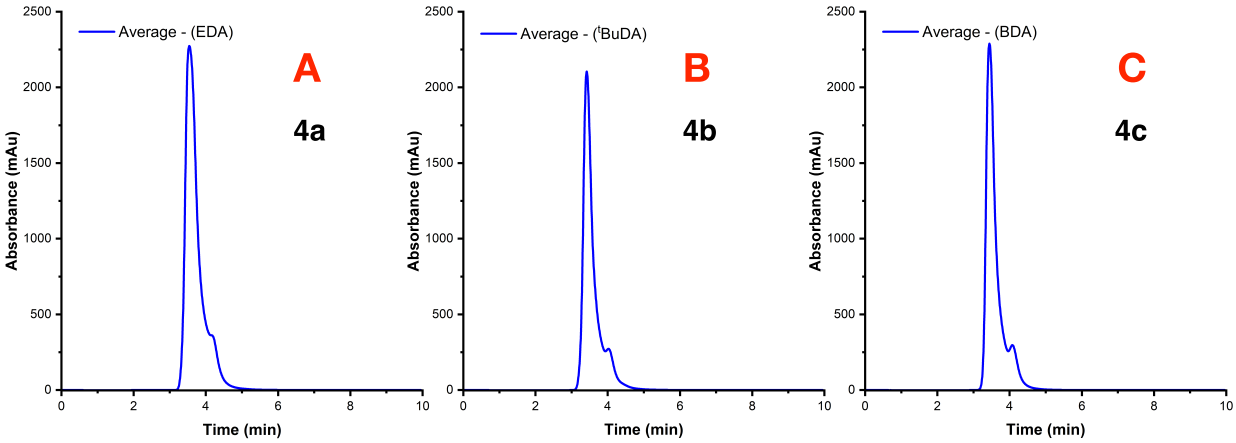

Figure S20. **Chiral-HPLC chromatograms for the C45-catalyzed N-H insertions reactions between *p*-chloroaniline and various diazo compounds.** Crude reaction mixtures for the products of the C45-catalyzed reaction between: *p*-chloroaniline (30 mM) and EDA (10 mM), product **3a** (panel A); *p*-chloroaniline (30 mM) and ^{t}BuDA (10 mM), product **3b** (panel B); *p*-chloroaniline (30 mM) and BnDA (10 mM), product **3c** (panel C). All reactions were performed under identical conditions (100 mM KCl, 20 mM CHES, pH 8.6, 5% EtOH) with 10 µM C45 (0.1% catalyst loading). The N-H insertion product from each assay was quenched with 3M HCl and extracted with 1.25 mL of hexane prior to loading onto the column. A polar organic mobile phase (100% MeCN: 0.1% v/v TFA:0.1% v/v: Et_3N) was employed and injection volumes were 2 µl. Chromatograms were recorded at 254 nm.These data were collected using a chiral-HPLC column (Astec CHIROBIOTIC®V, 250 x 21 mm, 5 µm), and for full experimental details see the *Materials and Methods* section.

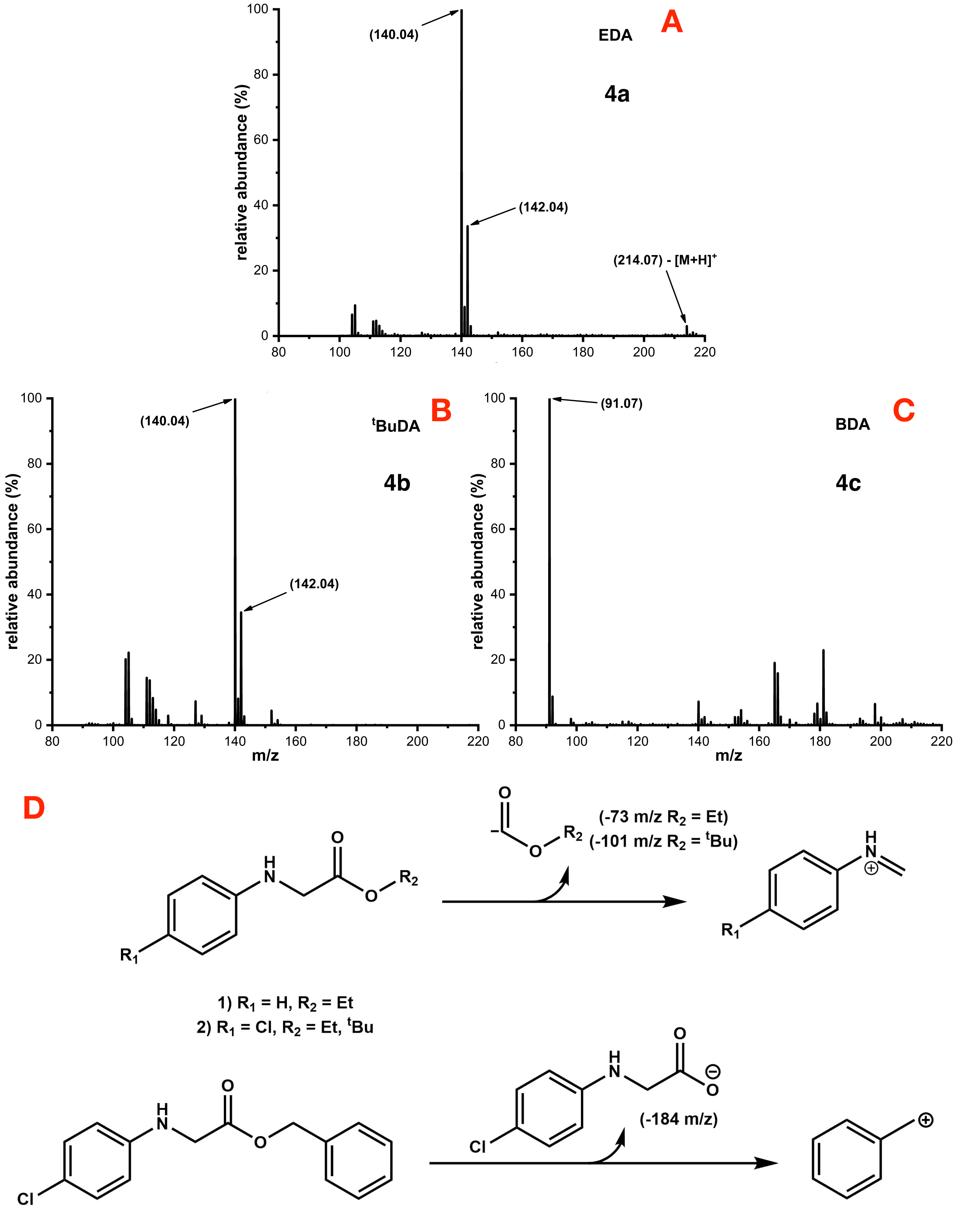

Figure S21. LC-MS spectra for the C45-catalyzed N-H insertions reactions between *p*-chloroaniline and various diazo compounds. A. C45-catalyzed N-H insertion assay between *p*-chloroaniline (30 mM) and EDA (10 mM), product **4a**. **B.** C45-catalyzed N-H insertion assay between *p*-chloroaniline (30 mM) and ^{t}BuDA (10 mM), product **4b**. **C.** C45-catalyzed N-H insertion assay between *p*-chloroaniline (30 mM) and BnDA (10 mM), product **4c**. All spectra were recorded in ES+ mode and monitored at 254 and 280 nm. A C8 column was employed for the LC separation with a gradient mobile phase (95:5:0.1% v/v water/ MeCN/formate 10:90:0.1% v/v water/MeCN/formate)**. D.** Assignment of major product peaks in the mass spectra. Where the $[M+H]^+$ ion is not observed, comparison of MS fragmentation patterns and HPLC elution profiles with synthesised, characterised standards are used to identify product.

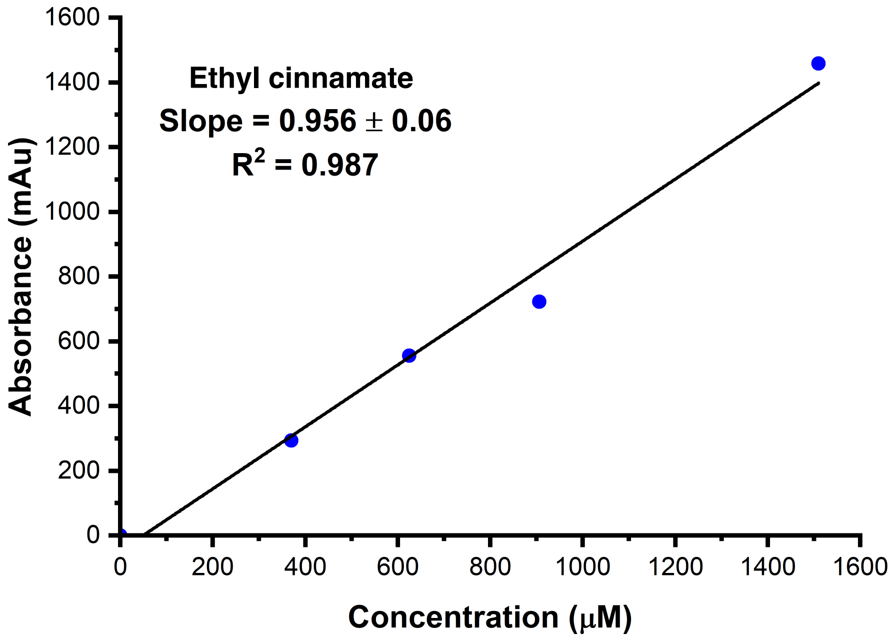

Figure S22. C18-HPLC external calibrations for ethyl cinnamate at 245 nm. A polar organic mobile phase (100% MeCN: 0.1% v/v TFA: 0.1% v/v: Et_3N) was employed and injection volumes were 8 µl. These calibrations were carried out on a C18 HPLC reverse phase column (Phenomenex, 150 x 15 mm, 5 µm).

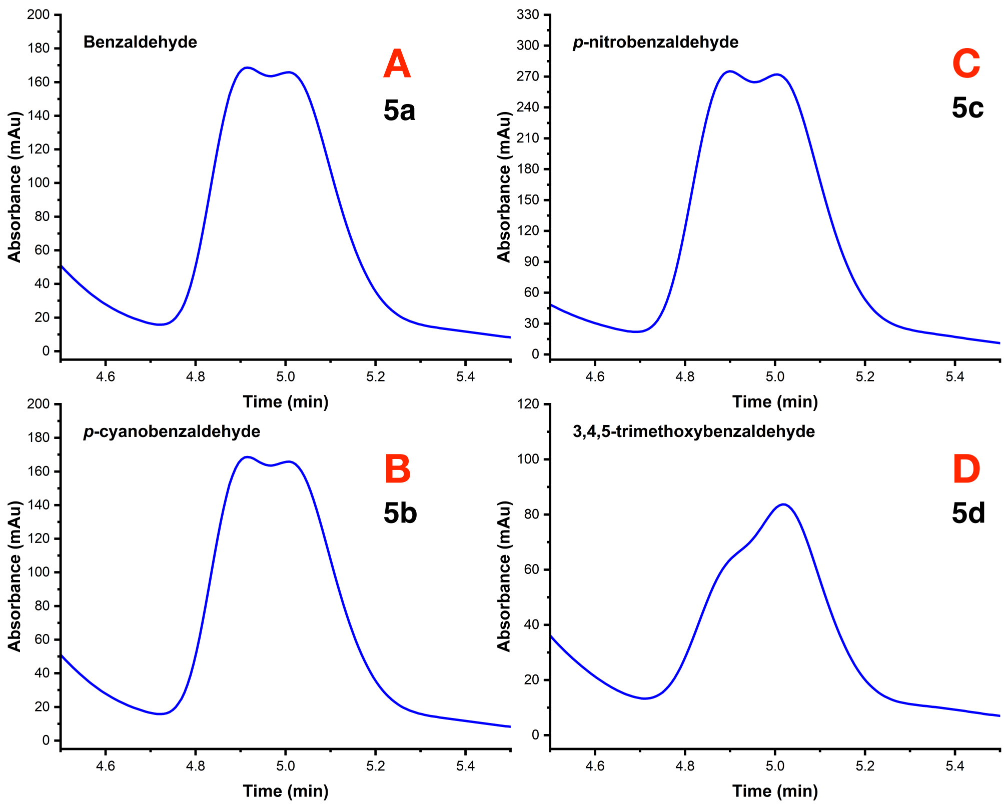

Figure S23. C18-HPLC chromatograms for the C45 (10 µM, 0.1% catalyst loading) catalyzed carbonyl olefination assays. A. benzaldehyde (10 mM) and EDA (10 mM), product **5a**; **B.** *p*-cyanobenzaldehyde (10 mM) and EDA (10 mM), product **5b**; **C.** *p*-nitrobenzaldehyde (10 mM) and EDA (10 mM), product **5c**; **D.** 3,4,5-trimethoxybenzalehyde (10 mM) and EDA (10 mM), product **5d.** All assays were performed in CHES buffer (pH 8.6) with 10 mM PPh_3 (in acetone). The α,β-unsaturated carbonyl product from each assay was extracted with 1 mL CH_2Cl_2 prior to loading onto the column. A polar organic mobile phase (100% MeCN: 0.1% v/v TFA: 0.1% v/v: Et_3N) was employed and injection volumes were 20 µl; all traces were recorded at 245 nm. The *E*-isomer eluted first and was followed by the *Z*-isomer. The relative peak heights for the *E* and *Z* isomers were used to calculate the cis/trans ratio using the equation [*E*]/[*Z*]. These data were collected using a C18 HPLC reverse phase column (Phenomenex, 150 x 15 mm, 5 µm), and for full experimental details see the *Materials and Methods* section.

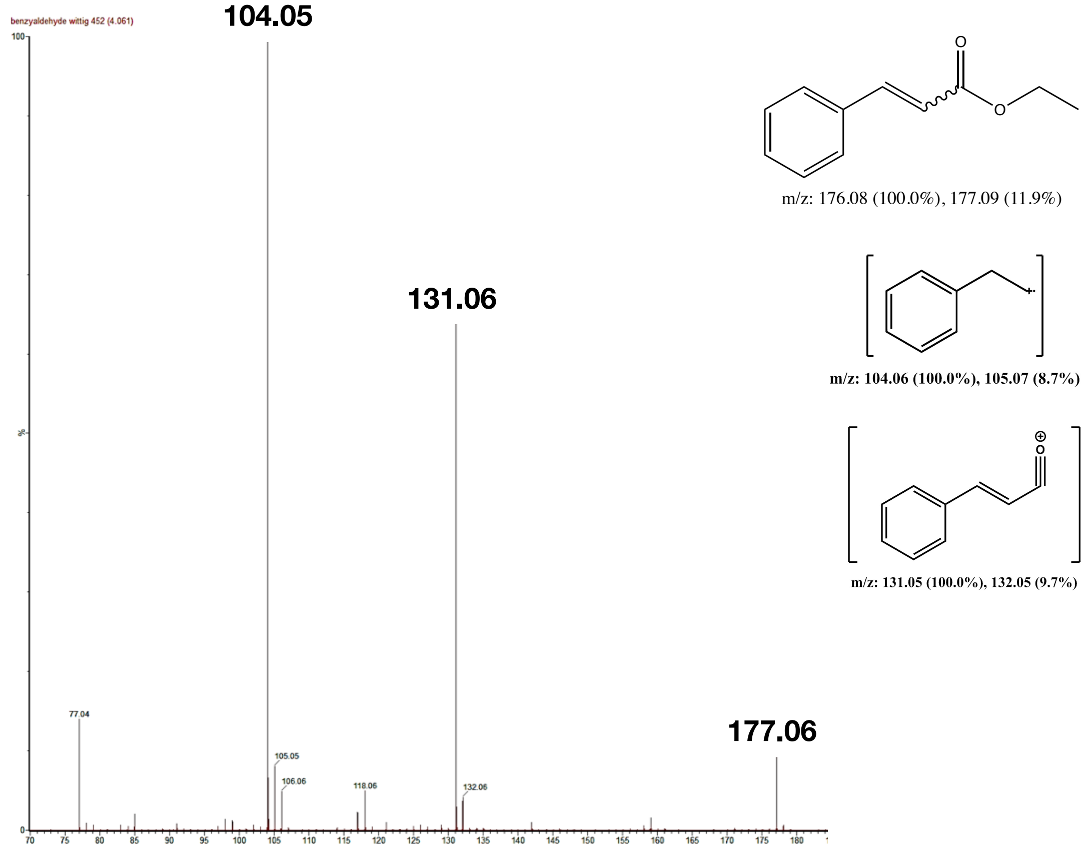

Figure S24. LC-MS spectrum of the C45 catalyzed carbonyl olefination assay products. LC-MS spectrum of the C45 catalyzed carbonyl olefination assay products. The mass spectrum was recorded in ES+ mode and monitored at 245 nm. A C8 column was employed for the LC separation with a gradient mobile phase (95:5% H_2O:MeCN to 10:90% H_2O:MeCN; 0.1% v/v formic acid, 0.25 mL min^{-1}).

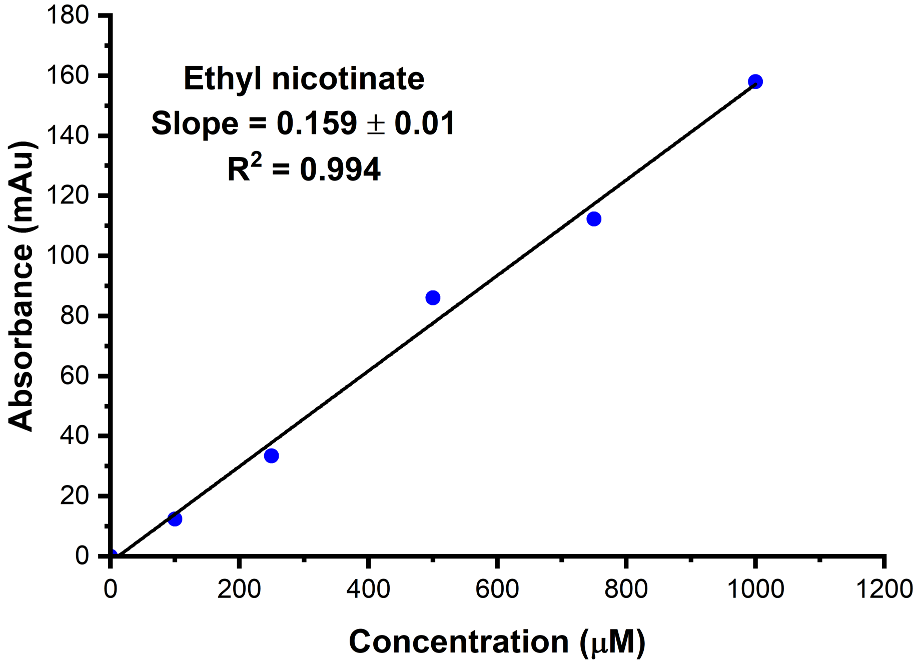

Figure S25. Reverse phase C18 HPLC external calibrations for nicotinate at 265 nm. A polar organic gradient was employed as the mobile phase (70:30% H2O:CH3CN to 10:90% H2O:CH3CN; 2 mL.min-1) and injection volumes were 8 µl. These calibrations were carried out on a C18 HPLC reverse phase column (Phenomenex, 150 x 15 mm, 5 µm).

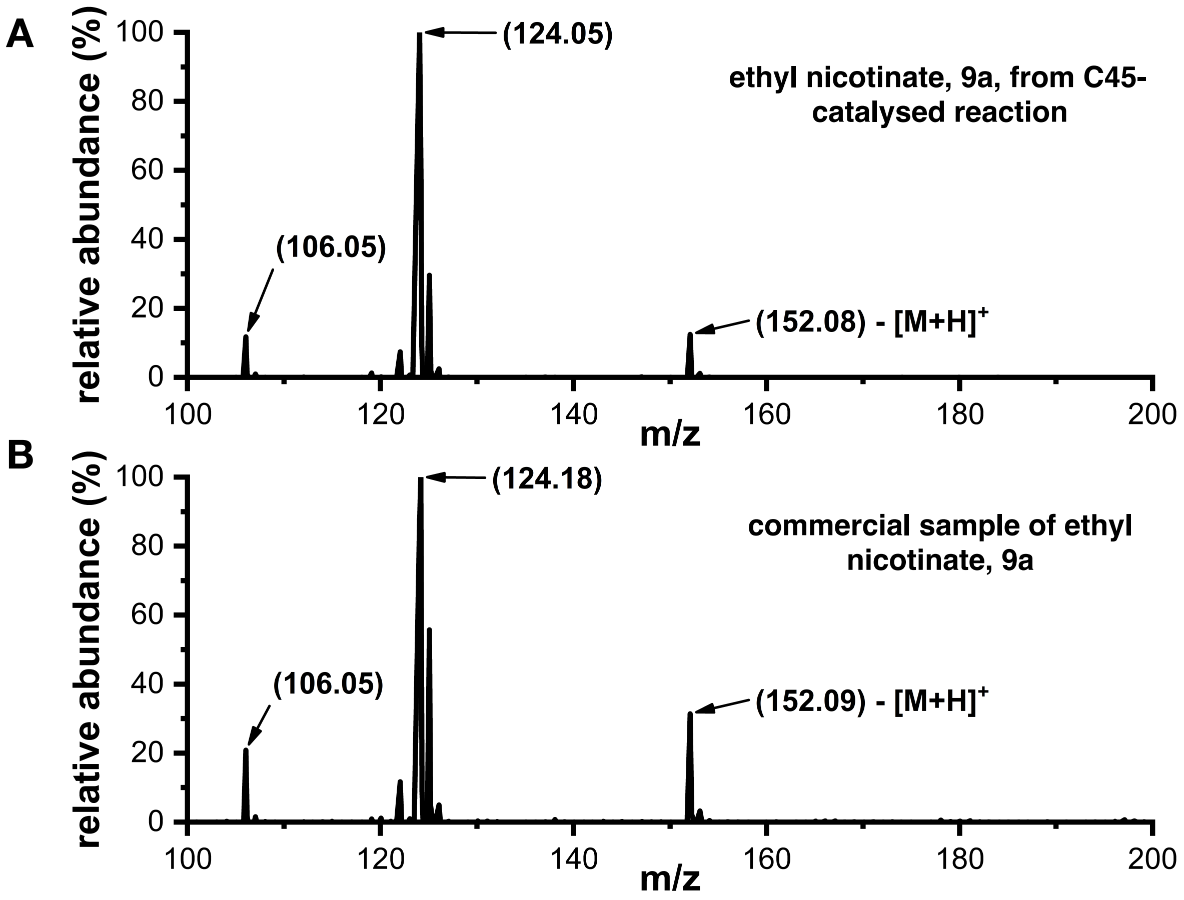

Figure S26. LC-MS spectra of C45 catalyzed ring expansion assay product. A. C45-catalyzed (10 µM, 1% catalyst loading) ring expansions assay between pyrrole (1 mM) and ethyl 2-bromo-2-diazoacetate (10 mM) to form ethyl nicotinate, product **9a**. **B.** a commercial sample of ethyl nicotinate, **9a**. All spectra were recorded in ES+ mode and monitored at 265. A C8 column was employed for the LC separation with a gradient mobile phase (95:5:0.1% v/v water/MeCN/formate 10:90:0.1% v/v water/MeCN/formate)**. C.** Assignment of major product peaks in the mass spectra.

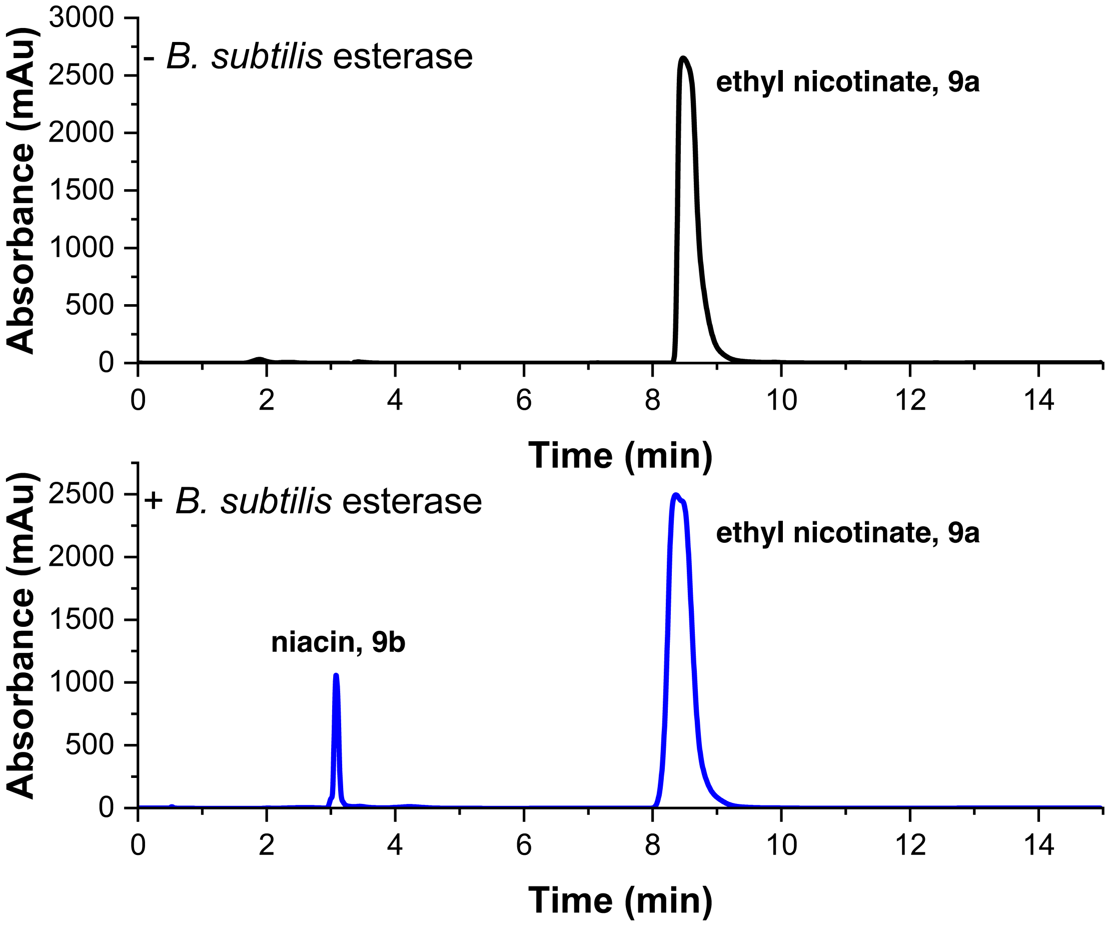

Figure S27. C18-HPLC chromatograms of ethyl nicotinate and esterase-hydrolyzed ethyl nicotinate. A. 50 mM commercial ethyl nicotinate, **9a**, (200 µl of 5 M stock in DMSO, 19.8 mL CHES buffer, pH 8.6) in the absence of esterase. **B.** 1 hour after the addition of 2 mg esterase (final esterase concentration is 100 µg/mL, 19.8 mL CHES buffer, pH 8.6). The mixture was analyzed directly after precipitating the esterase with 3 M trichloroacetic acid. A reverse phase gradient mobile phase (70:30% H_2O:MeCN to 10:90% H_2O:MeCN; 2 mL.min^{-1}) was employed and injection volumes were 20 µl; traces were recorded at 265 nm. These data were collected using a C18 HPLC reverse phase column (Phenomenex, 150 x 15 mm, 5 µm), and for full experimental details see the *Materials and Methods* section.

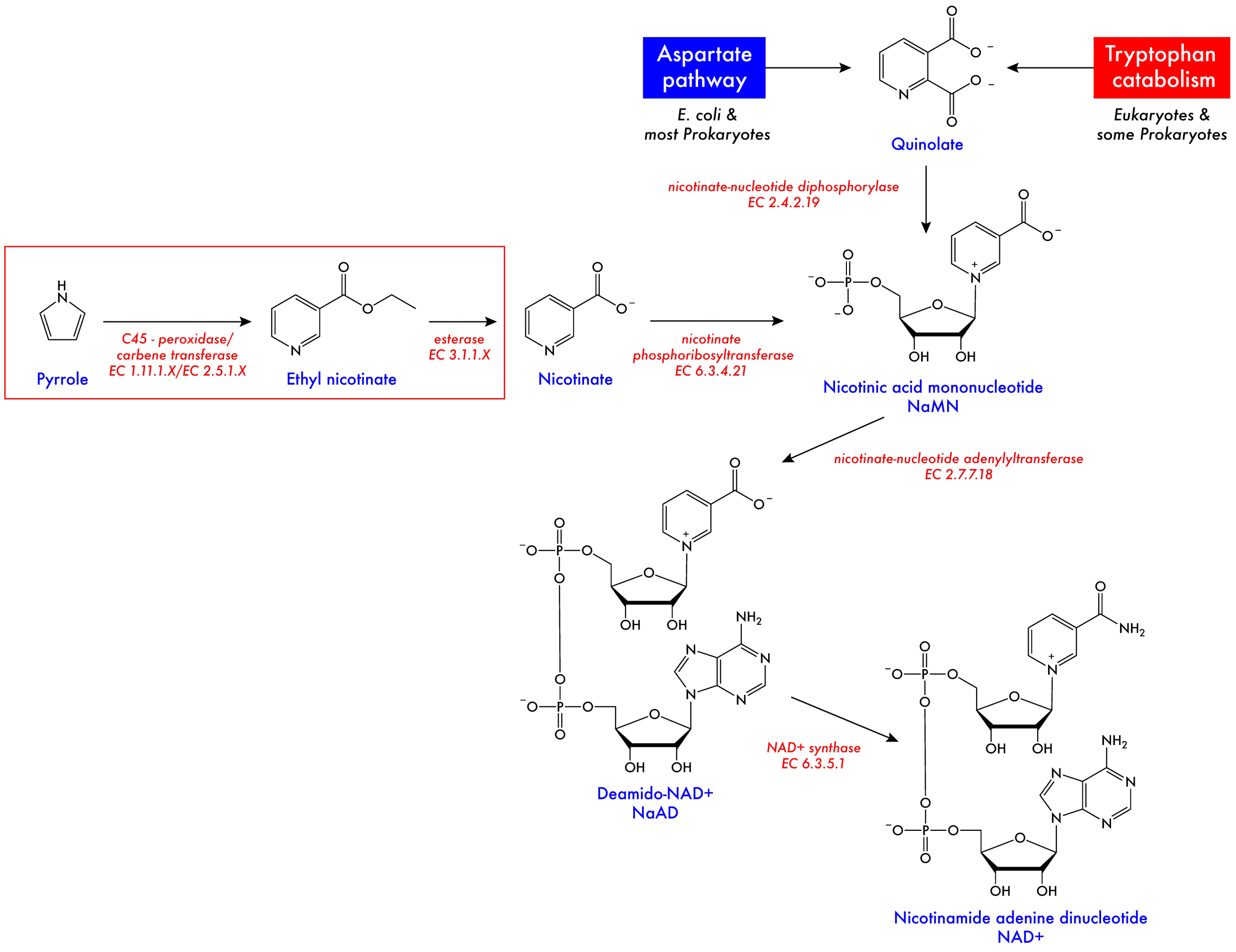

Figure S28. Natural and engineered biosynthetic pathways to NAD+. Steps catalyzed by the *de novo*-designed enzyme C45 and the non-native esterase from *B. subtilis* are displayed in the red box, showing an alternative route from pyrrole to nicotinate. We propose that deleting the *E. coli* nicotinate-nucleotide diphosphorylase and growing esterase- and C45-expressing cells under nicotinate starved conditions with added 2-bromo-2-diazoacetate would result in the life-sustaining biosynthesis of NAD+. Data and annotations in the figure were obtained from the KEGG database (https://www.genome.jp/kegg/kegg1.html) and the NC-IUBMB database (http://www.sbcs.qmul.ac.uk/iubmb/enzyme/).

Table S1. C45-catalyzed olefination of aldehydes.

Product	R1	R2	R3	% Yield (*cis*)	% Yield (*trans*)	% Yield (*cis* + *trans)*	*cis/ trans*	TTN (*cis*)	TTN (*cis* + *trans*)
**5a**	H	H	H	2.57 (±0.52)	2.37 (±0.24)	4.94	1.2:1	26	49.4
**5b**	H	CN	H	4.21 (±1.98)	4.21 (±1.98)	8.41	1:1.02	42	84.1
**5c**	H	N_2O	H	3.16 (±0.96)	3.19 (±1.00)	6.35	1:1	32	63.5
**5d**	OMe	OMe	OMe	5.47 (±3.91)	5.53 (±4.00)	11.0	1:1	55	110
